# SslE Elicits Functional Antibodies That Impair *In Vitro* Mucinase Activity and *In Vivo* Colonization by Both Intestinal and Extraintestinal *Escherichia coli* Strains

**DOI:** 10.1371/journal.ppat.1004124

**Published:** 2014-05-08

**Authors:** Barbara Nesta, Maria Valeri, Angela Spagnuolo, Roberto Rosini, Marirosa Mora, Paolo Donato, Christopher J. Alteri, Mariangela Del Vecchio, Scilla Buccato, Alfredo Pezzicoli, Isabella Bertoldi, Lapo Buzzigoli, Giovanna Tuscano, Maria Falduto, Valentina Rippa, Yaqoub Ashhab, Giuliano Bensi, Maria Rita Fontana, Kate L. Seib, Harry L. T. Mobley, Mariagrazia Pizza, Marco Soriani, Laura Serino

**Affiliations:** 1 Novartis Vaccines and Diagnostics Srl, Siena, Italy; 2 Department of Microbiology and Immunology, University of Michigan Medical School, Ann Arbor, Michigan, United States of America; 3 Biotechnology Research Center, Palestine Polytechnic University, Hebron, Palestine; 4 Institute for Glycomics, Griffith University, Gold Coast Campus, Queensland, Australia; University of Texas Southwestern Medical Center, United States of America

## Abstract

SslE, the Secreted and surface-associated lipoprotein from *Escherichia coli*, has recently been associated to the M60-like extracellular zinc-metalloprotease sub-family which is implicated in glycan recognition and processing. SslE can be divided into two main variants and we recently proposed it as a potential vaccine candidate. By applying a number of *in vitro* bioassays and comparing wild type, knockout mutant and complemented strains, we have now demonstrated that SslE specifically contributes to degradation of mucin substrates, typically present in the intestine and bladder. Mutation of the zinc metallopeptidase motif of SslE dramatically impaired *E. coli* mucinase activity, confirming the specificity of the phenotype observed. Moreover, antibodies raised against variant I SslE, cloned from strain IHE3034 (SslE_IHE3034_), are able to inhibit translocation of *E. coli* strains expressing different variants through a mucin-based matrix, suggesting that SslE induces cross-reactive functional antibodies that affect the metallopeptidase activity. To test this hypothesis, we used well-established animal models and demonstrated that immunization with SslE_IHE3034_ significantly reduced gut, kidney and spleen colonization by strains producing variant II SslE and belonging to different pathotypes. Taken together, these data strongly support the importance of SslE in *E. coli* colonization of mucosal surfaces and reinforce the use of this antigen as a component of a broadly protective vaccine against pathogenic *E. coli* species.

## Author Summary

*Escherichia coli* are the predominant facultative anaerobe of the human colonic flora. Although intestinal and extraintestinal pathogenic *E. coli* are phylogenetically and epidemiologically distinct, we recently proposed a number of protective antigens conserved in most *E. coli* pathotypes. In this study, we have elucidated the function of the most promising of these antigens, SslE, which is characterized by the presence of a M60-like domain representative of a new extracellular zinc-metalloprotease sub-family. In particular, *in vitro* analysis of the ability of an *sslE* knockout mutant strain to transverse an agar-based mucin matrix revealed that SslE is essential to *E. coli* mucinase activity. Evidence showing that SslE induces functional antibodies, preventing both *in vitro* mucin degradation but also *in vivo* gut, kidney and spleen colonization, further support the hypothesis that SslE may facilitate *E. coli* colonization by favoring the penetration of the sterile inner mucus layer leading to interaction with host cells. Finally, the ability of SslE to also induce protective immunity against sepsis, linked to its presence among different pathotypes, supports the use of such an antigen as a broadly protective *E. coli* vaccine candidate.

## Introduction

Pathogenic *E. coli* can be broadly classified as either extraintestinal pathogenic *E. coli* (ExPEC), the main cause of urinary tract infection (UTI), newborn meningitis and sepsis, or as intestinal pathogenic *E. coli* (InPEC) causing diarrhoeagenic infections. Among the intestinal pathogens there are at least six well-described groups: enteropathogenic *E. coli* (EPEC), enterohaemorrhagic *E. coli* (EHEC), enterotoxigenic *E. coli* (ETEC), enteroaggregative *E. coli* (EAEC), enteroinvasive *E. coli* (EIEC) and diffusely adherent *E. coli* (DAEC) [1]. The plasticity of the *E. coli* genomes, due to the ability to gain or lose virulence attributes by horizontal gene transfer, allows these organisms to colonize different sites. Indeed, *E. coli* possesses an array of virulence factors which include various adhesins, capsule, iron-transporters, toxins and proteases (reviewed in [1]). However, recent studies have suggested that the pathogenesis of *E. coli* is considerably more complex than previously appreciated involving additional virulence factors [2], [3]. The absence of a broadly protective vaccine against pathogenic *E. coli* is a major problem for modern society since diseases caused by these bacteria are associated with significant human suffering and high healthcare costs. The overall problem is exacerbated by the rising rates of multi-drug resistant strains and by the emergence of new sequence types and hypervirulent strains [4]–[9]. We have recently proposed ECOK1_3385 as a promising vaccine candidate able to confer protection in a murine model of sepsis [10], [11]. This protein, described as SslE (for secreted and surface-associated lipoprotein from *E. coli*) and formerly known as YghJ [12], [13], appears to be required for biofilm formation and for virulence of EPEC strains [14], although more recent evidence indicates that SslE has no effect on adherence and biofilm formation in atypical EPEC strains [15]. Thus, the function of SslE remains to be fully elucidated. However, it is known that SslE is secreted through a type II secretion system (T2SS), an exporting apparatus typically used by Gram-negative bacteria to secrete virulence determinants [16]. Two T2SSs exist in *E. coli*, designated as alpha (T2SSα) and beta (T2SSβ) [17]. The T2SSβ operon is composed of three genes (*yghJ*, *pppA*, and *yghG*) upstream of *gspC*β. The first gene, *yghJ*, encodes for the SslE protein. A functional T2SSβ secreting a cognate SslE protein was recently studied in the non-pathogenic *E. coli* W strain [18]. Recently, it was reported that SslE belongs to a new sub-family of extracellular zinc-metallopeptidases, characterized by a M60-like zinc-metalloprotease domain HEXXHX(8,24)E [19], that is distantly related to known viral enhancin zinc-metallopeptidases. The baculovirus enhancin protein Vef is able to digest intestinal mucins, facilitating the attachment and entry of the virus into epithelial cells [20].

Using biochemical and functional assays, we demonstrated that SslE is involved in *E. coli* degradation of mucin substrates. In addition, antibodies raised against SslE variant I from ExPEC strain IHE3034 were able to inhibit translocation of different *E. coli* pathotypes through a mucin-based matrix, suggesting a possible mechanism for *in vivo* protection. This hypothesis was corroborated by the fact that in mouse models of intestinal and urinary tract colonization, SslE variant I induced protective immunity also against *E. coli* strains expressing variant II. The widespread distribution and conservation of SslE, together with the ability to elicit functional antibodies, assessed both *in vitro* and *in vivo*, strongly support the potential of the SslE antigen to provide coverage against both intestinal and extraintestinal pathogenic *E. coli* strains.

## Results

### SslE localizes on *E. coli* surface at distinct foci

**Figure 1:**
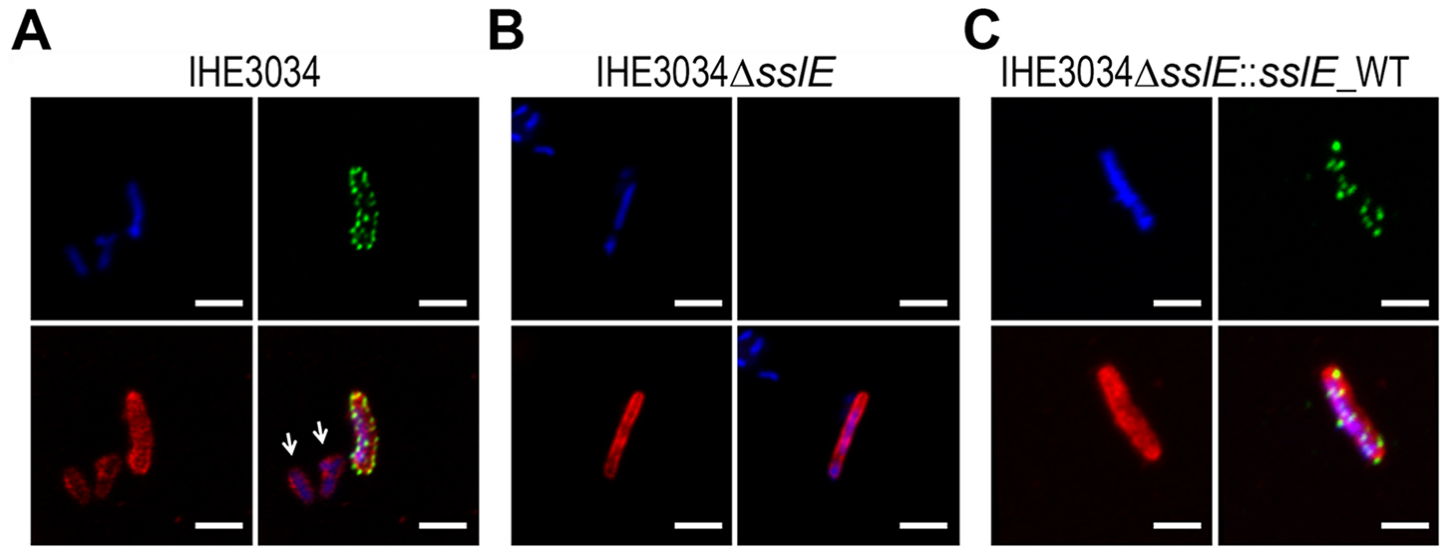
SslE surface localization on the ExPEC strain IHE3034. Confocal images of (A) IHE3034 wild-type, (B) IHE3034Δ*sslE* knockout mutant and (C) IHE3034Δ*sslE*::*sslE*_WT complemented strain. SslE was detected using specific anti- SslE antibodies raised in rabbits and visualized using a fluorescent secondary antibody (green). Antibodies to whole-IHE3034 bacteria and a fluorescent secondary antibody (red) and DAPI (blue) were used to visualize bacteria and chromosomal DNA, respectively. White arrows indicate bacteria negative for SslE staining. Bars, 1 µm.

It has been recently reported that although SslE is secreted by a T2SS, it is also found on the bacterial cell surface [10], [14]. Confocal analysis of Z-stack images of an ExPEC strain IHE3034 stained for SslE and deconvoluted using Volocity Software, revealed that the antigen is translocated on the bacterial surface at specific foci (Fig. 1A). Of interest, we observed that only a small proportion of bacteria (3% of total number) expressed the antigen on the surface (Fig. 1A). We determined that this phenotype is attributable to the polysialic acid capsule (K1 antigen) that it responsible for masking SslE on the bacterial surface (Fig. S1). The *sslE* deletion mutant strain (IHE3034Δ*sslE*) did not show any surface labeling (Fig. 1B), confirming the specificity of the signal. Complementation of the mutant strain with a pET24b+ plasmid carrying the *sslE* gene (including the promoter region) restored antigen surface localization (Fig. 1C). To exclude the possibility that the SslE signal at the bacterial surface could be partially attributed to the re-association of the secreted form of the protein to the membrane, we co-cultured the IHE3034 wild-type (WT) strain with the IHE3034Δ*sslE* strain engineered to express the GFP fluorescent protein. Staining of bacteria using SslE antibodies conjugated to FITC, revealed that the antigen was exclusively detected on the surface of the WT strain and not on the fluorescent bacteria, indicating that no SslE re-association occurred (data not shown).

### SslE is involved in *in vitro* mucin degradation by IHE3034 strain

**Figure 2:**
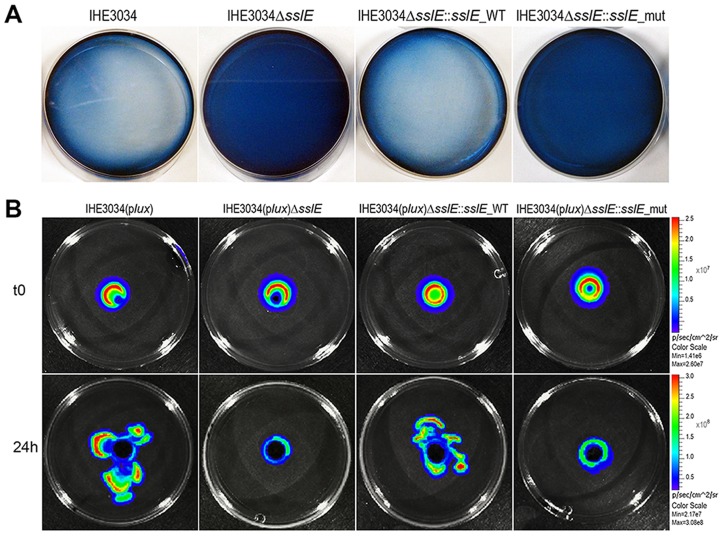
SslE mucinolytic activity. (A) Mucin lysis (clear plates) was assessed by amido black staining. IHE3034 wild-type, IHE3034Δ*sslE* knockout mutant, IHE3034Δ*sslE*::*sslE_*WT (complemented with the *sslE* wild-type gene), and IHE3034Δ*sslE*::*sslE_*mut (complemented with the *sslE* gene mutated in the putative metallopeptidase motif), were grown on plates containing 0.5% bovine submaxillary mucin (SIGMA) and stained with 0.1% (wt/vol) amido black in 3.5 M acetic acid for 30 min and destained with 1.2 M acetic acid. (B) The four strains were engineered for constitutive luciferase expression (p*lux* operon) and mucinolytic activity was detected by the *In Vivo* Imaging System (IVIS) technology. Bacterial migration in the mucin-agar plates is shown, from the point of inoculum (time zero; t0) to growth at 24 hours (24 h).

As recently reported by Nakjang and collaborators [19], HEXXHX(8,24)E is the full putative metalloprotease core motif of SslE (residues: 1304–1322; SslE accession number: YP_006102500), exclusively present in a recently characterized zinc metallopeptidase sub-family possessing mucinase activity [19]. The pattern “HEXXHX(8,24)E” consists of a conserved glutamate residue localized 8 to 24 amino acids from the “HEXXH” motif. To investigate the putative mucinolytic activity of SslE, we have applied a number of *in vitro* assays previously reported to specifically detect mucinase activity in bacteria [21]–[24]. The first approach is based on the use of bacteria grown on agar plates containing 0.5% bovine submaxillary mucin followed by amido black-staining [25], [26]. Plates containing the IHE3034 WT strain incubated for 24 h revealed clear zones of mucin lysis (Fig. 2A). However, no cleared areas were detected when the IHE3034Δ*sslE* knockout (KO) strain was added to the plates, indicating the specific contribution of SslE to the mucinase activity. Incubation of mucin-based plates with the complemented strain IHE3034Δ*sslE::sslE*_WT carrying the WT *sslE* gene fully restored the wild-type phenotype as assessed by the lack of amido black staining. To investigate the role of the M60-like core motif in mucin lysis, we transformed the IHE3034Δ*sslE* strain with the pET24b+ plasmid carrying a triple mutation in the putative metallopeptidase motif of SslE (**YV**VG**Y** vs. HEVGH). In particular, we introduced hydrophobic elements in the HEXXH motif (Y and V), which by reducing the charge of the enzymatic task are likely to impair the mucinase activity. Testing of this mutant by the amido black assay revealed a phenotype comparable to the *sslE* KO strain (Fig. 2A). These data were further confirmed by the *In Vivo* Imaging System (IVIS-Perkin Elmer) technology which allowed the visualization of bacterial migration through the agar-mucin matrix at different time points, using IHE3034 strains engineered for constitutive expression of a luciferase operon (p*lux*) [27] (Fig. 2B). Briefly, a mid-log bacterial culture of the bioluminescent strains was loaded in a well created at the center of a mucin-agar plate and bacterial distribution was detected after 24 h of incubation. IHE3034(p*lux*) and IHE3034(p*lux*)Δ*sslE::sslE*_WT strains, but not IHE3034(p*lux*)Δ*sslE* and IHE3034(p*lux*)Δ*sslE::sslE*_mut, were able to spread beyond the site of the initial inoculum (Fig. 2B), confirming that SslE-dependent mucin degradation enables diffusion of *E. coli* through the agar.

### Antibodies against SslE prevent the ability of IHE3034 to cross a mucin-based matrix in a dose-dependent manner

**Figure 3:**
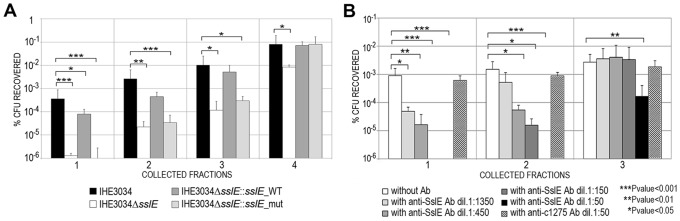
Anti-SslE antibodies impair translocation of *E. coli* through a mucin-gel matrix. (A) IHE3034 wild-type, the IHE3034Δ*sslE* knockout mutant, IHE3034Δ*sslE*::*sslE_*WT (complemented with the *sslE* gene wild-type) and IHE3034Δ*sslE*::*sslE_*mut (complemented with *sslE* gene mutated in the putative metallopeptidase motif), were loaded on the top of a mucin-gel matrix column polymerized in a 1 ml syringe. After 3 hours at 37°C, eluted fractions were collected and plated for CFU counting. The results were reported as percentage of CFU recovered in four different fractions, sequentially eluted from the bottom of the column, with respect to the initial inoculum. (B) Dose-dependent inhibition of IHE3034 translocation through a mucin-gel matrix by anti-SslE antibodies. Serial dilutions (range 1∶50–1∶1350) of antibodies were used for inhibition. Antibodies against the unrelated ExPEC c1275 were used as a negative control at the dilution 1∶50. Translocation was reported as the percentage of CFU recovered with respect to the initial inoculum for three sequentially eluted fractions. P values were determined using a two-tailed unpaired Student's significance test.

To test the hypothesis that anti-SslE antibodies may also inhibit mucinase activity *in vitro*, we developed an *in vitro* system to quantify the ability of strain IHE3034 WT to transverse a mucin-based gel matrix. An agar-based matrix gel containing 10% bovine submaxillary gland mucins was reconstituted in a 1 mL syringe and bacterial aliquots (10^8^ CFU) were layered on top of the gel and statically incubated for 3 h at 37°C in a vertical position to allow bacterial translocation. At the end of the incubation period, gel fractions were eluted from the bottom of the syringe, diluted and plated for CFU determination. After confirming the impaired phenotype of the *sslE* KO strain in traversing the mucin matrix compared to the isogenic WT strain (∼2.5 Log reduction) (Fig. 3A), we tested the ability of polyclonal antibodies generated by sub-cutaneous immunization of rabbit with the full length recombinant SslE from ExPEC strain IHE3034 (anti-SslE_IHE3034_) to reduce bacterial translocation through the mucin agar-gel syringe. Anti-SslE IgGs and IgAs in the rabbit serum were measured by ELISA (Fig. S2A and B). A significant dose-dependent inhibition of bacterial translocation was observed when the mucin-gel matrix was polymerized together with anti-SslE antibodies (dose range 1∶50 to 1∶1350) (Fig. 3B). At a dilution of 1∶50 the inhibitory effect of SslE antibodies was evident in all fractions collected, while higher dilutions principally affected bacterial translocation in the first two fractions. A higher dilution of 1∶4050 did not show an inhibitory effect in any of the collected fractions (data not shown). The specificity of the inhibition was confirmed by the absence of an effect when using an antiserum against the unrelated ExPEC antigen c1275 [10], at the lowest dilution (Fig. 3B). On the other hand, since antibodies against a fragment of SslE, C-SslE_IHE3034_, lacking the M60-like motif (Fig. S3A), were still capable of impairing IHE3034 translocation through the mucin layer (Fig. S3B), it is not possible to establish whether polyclonal antibodies have a direct or an indirect effect on SslE activity.

### Antibodies against variant I SslE from IHE3034 inhibit *E. coli* mucin translocation in strains expressing variant II SslE

**Figure 4:**
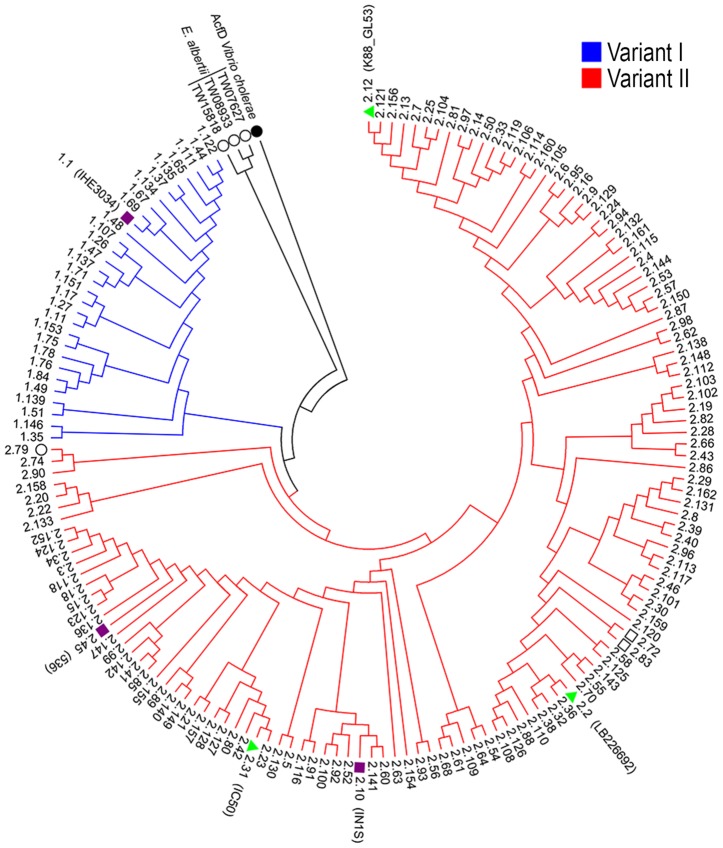
Phylogenetic tree of SslE from a panel of *E. coli* isolates. The phylogenetic tree of 155 unique *E. coli* SslE protein sequences was inferred using the neighbor-joining method. Two main SslE variants are highlighted with different colors: blue for variant I and red for variant II. Purple bullets refer to ExPEC and green triangles to InPEC isolates used in experiments. Black circles indicate SslE amino acid homologues of *Vibrio cholerae* and *Escherichia albertii* used as outgroup sequences. The tree also includes two sequences from *Escherichia fergusonii* (black square). The strains are designed with patterns made by a first number relative to the SslE main variant and a second number corresponding to the sub-variant (see table S1 and table S2 for immediate identification). Phylogenetic analysis were conducted by MEGA4 software [52].

**Figure 5:**
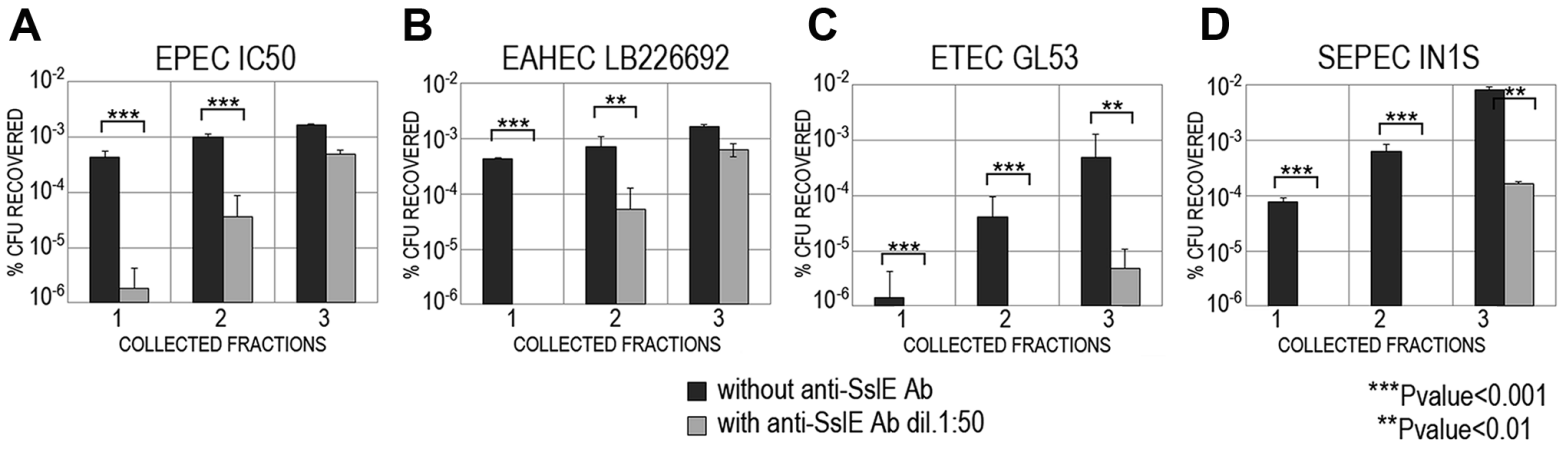
Cross-inhibition of *E. coli* translocation through a mucin-gel matrix by anti-SslE_IHE3034_ (belonging to variant I) antibodies. EPEC IC50 (A), EAHEC LB226692 (B), ETEC GL53 (C), SEPEC IN1S (D) strains carrying variant II were loaded on top of the gel-mucin matrix column and bacterial translocation with or without anti-SslE_IHE3034_ antibodies was assessed in three sequentially collected fractions. For each strain, translocation was reported as the percentage of CFU recovered with respect to the initial inoculum.

As previously reported, SslE can be divided into two main variants [10]. Three hundred and eighteen *E. coli sslE* sequences were added to the 96 previously analyzed by Moriel *et al.* [10] (Table S1) and global amino acid sequence alignment revealed that sequence variability was present and distributed along the entire protein sequence. Overall, amino acid sequence identity ranged from 86–100%, with the HEXXHX(8,24)E core motif fully conserved in all sequences analysed. A total of 155 *E. coli* unique protein sequences were identified and used to construct a phylogenetic tree (Fig. 4 and Table S2). The two main branches denoted the presence of two SslE clades (encoding for two variants: I and II). To understand whether antibodies raised against variant I can cross-inhibit the mucinolytic activity of other SslE sub-variants, we selected a number of strains producing SslE variant II and belonging to different pathotypes. We tested the ability of an antiserum against SslE from strain IHE3034 (SslE_IHE3034_, belonging to variant I) to prevent the translocation of intestinal and extraintestinal strains expressing SslE belonging to variant II. In particular, we selected an EPEC strain (IC50), a SEPEC (septicemic-associated *E. coli* belonging to ExPEC) strain (IN1S), an ETEC strain (GL53) and the EAHEC strain (LB226692) recently identified to be responsible for the 2011 German *E. coli* outbreak. SslE_IHE3034_ antiserum inhibited the ability of all *E. coli* pathotypes tested (expressing SslE variant II) to traverse the mucin-based matrix (Fig. 5).

### SslE promoter is functional in a mouse model of intestinal colonization

**Figure 6:**
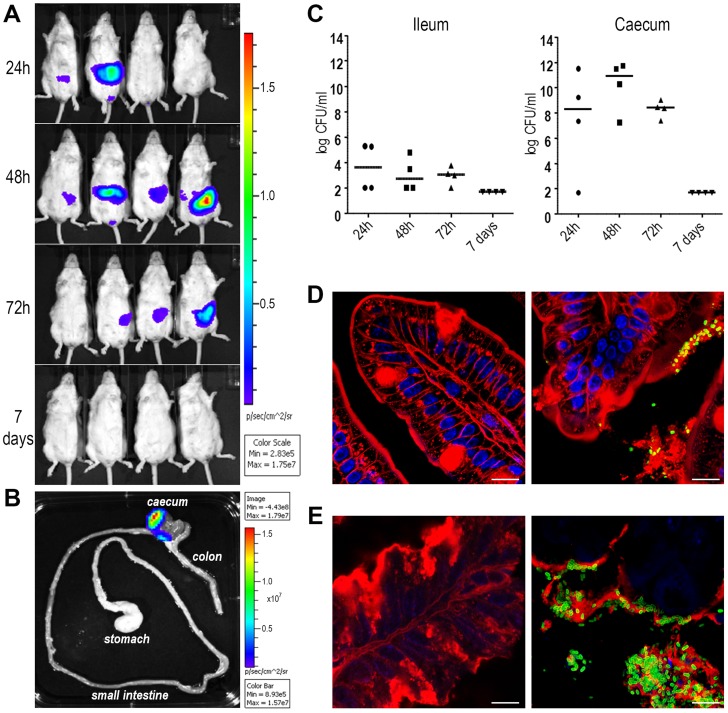
ETEC strain GL53 is able to colonize the mouse intestine. (A) A group of 4 mice was infected with bioluminescent GL53 strain and monitored up to 7 days by IVIS System. The data displayed illustrate the results of a representative experiment. (B) Distribution of the bioluminescent GL53 strain in the intestinal tract of infected mice shows bacterial accumulation in the caecum. (C) Quantitative analysis of intestinal colonization by GL53. Briefly, ileum and caecum at 24 h, 48 h, 72 h and 7 days post infection were homogenized and plated for CFU counts. Symbols represent single mice and the median is shown as bars for each time point. (D) Confocal staining of uninfected (left panel) and GL53 infected (right panel) ileum and (E) images of uninfected (left panel) and infected caecum (right panel). Tissues were visualized with the red fluorescent Wheat Germ Agglutinin (Alexa Fluor 568-WGA, Life Technologies) and nuclei with the blue fluorescent DAPI. Bacteria were detected using polyclonal antibodies against GL53 and visualized by green Alexa Fluor 488-conjugated secondary antibody (Life Technologies). Bars: 10 µm.

**Figure 7:**
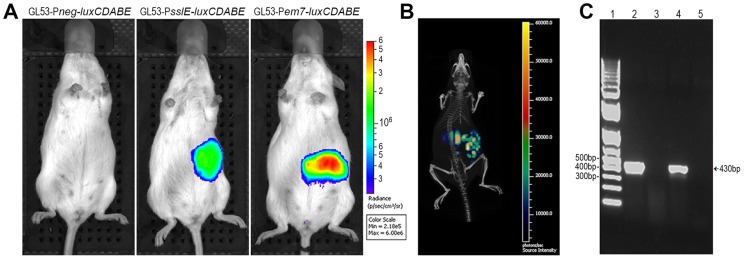
The *sslE* promoter is functional in an intestinal model of colonization. (A) 2D *in vivo* imaging at 24 hours of mice intragastrically infected with GL53-P*neg*-*luxCDABE* (promoterless control vector), with the bioluminescent derivative GL53-P*sslE*-*luxCDABE* and with the GL53-P*em7-luxCDABE* (positive control). (B) 3D image reconstruction showing *ssIE*-promoter driven luciferase expression in *E. coli* localized in the intestinal tract. (C) RT-PCR of RNA purified from: *in vitro* lab-grown GL53 bacteria (lane 2, positive control); caecum tract of uninfected mice (lane 3, negative control); GL53 bacteria recovered from infected mice (lane 4); GL53 bacteria recovered from infected mice without the RT step (lane 5). 1 Kb Plus DNA Ladder (Life Technologies) is shown in lane 1.

The evidence that antibodies against SslE_IHE3034_ are functional and recognize different variants allows us to postulate that a vaccine containing this antigen may have the potential to protect against most pathogenic *E. coli* species. In order to test the protective efficacy of SslE_IHE3034_ (variant I), we set up a mouse model of intestinal colonization using the ETEC GL53 strain. Mice were intragastrically infected with the bioluminescent GL53-P*em7-luxCDABE* strain [27] and consistent bioluminescent signals were detected in the abdominal region until to 72 hours post-infection by the *In Vivo* Imaging System (IVIS) (Fig. 6A). As observed for other intestinal *E. coli* pathotypes [28], [29], bacterial infection mainly occurs in the caecum tract (Fig. 6B). This is consistent with data obtained by both CFU counts from infected intestinal ileum and caecum tracts (Fig. 6C) and confocal imaging of tissues (Fig. 6D and E). After setting up the GL53 intestinal colonization, we evaluated the functionality of the *sslE* promoter *in vivo*. 2D bioluminescent signal in the abdominal region could be observed when the luciferase expression was driven by the *sslE* promoter (Fig. 7A), compared to the positive control GL53-P*em7-luxCDABE*. As expected, GL53 transformed with the luciferase promoterless plasmid gave no signal (Fig. 7A) [27]. In addition, 3D analysis confirmed that the signal was predominantly associated with the intestine (Fig. 7B). *SslE* transcription in GL53 colonizing bacteria was further evaluated by reverse transcriptase-polymerase chain reaction (RT-PCR), confirming that the *sslE* promoter is active *in vivo* (Fig. 7C).

### Variant I SslE induces cross-protective immunity against variant II SslE expressing strains in intestinal colonization, UTI and sepsis mouse models

**Figure 8:**
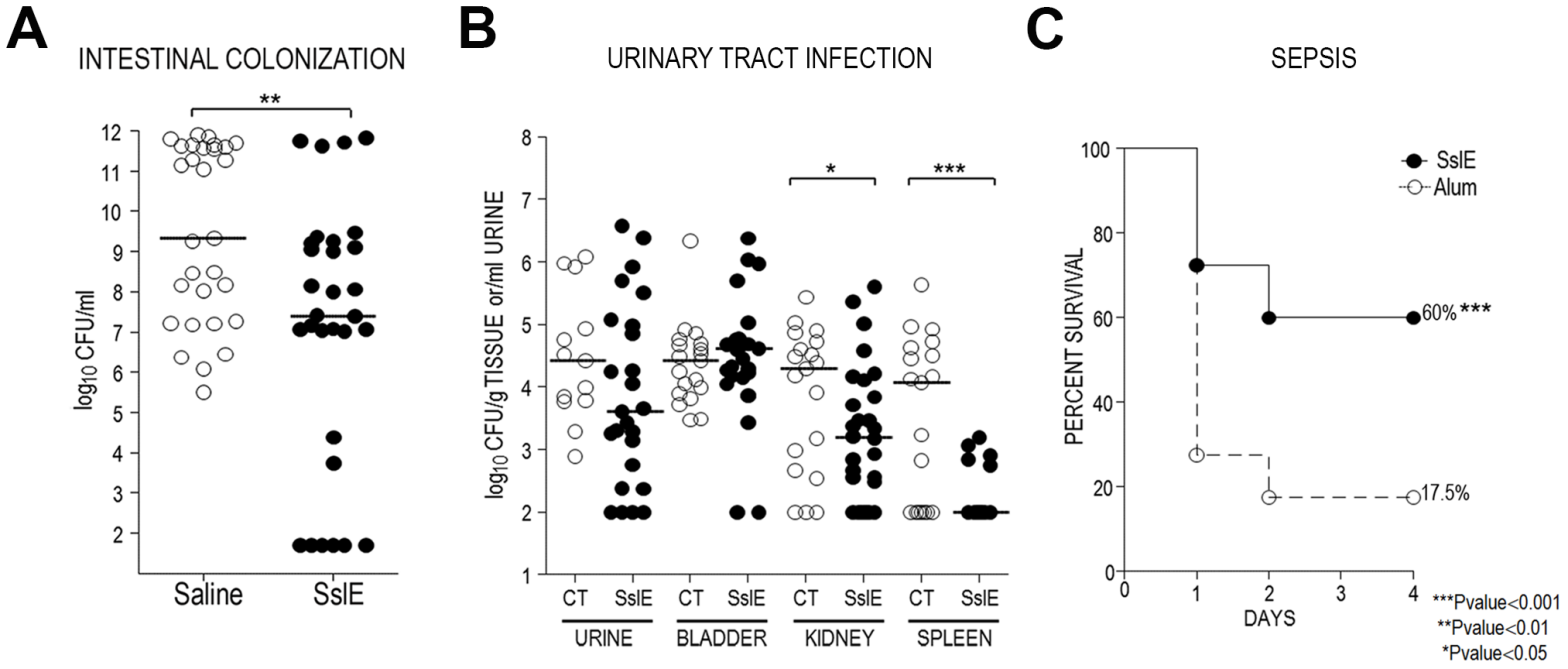
SslE_IHE3034_ induces cross-protection in intestinal colonization, UTI and sepsis models. (A) Thirty CD1 mice were intranasally immunized with 30 µg of SslE_IHE3034_ at days 1, 21 and 35. Saline was used in the negative control groups. Challenge was done by oral gavage with 5×10^7^ CFU of strain GL53 at day 49. Serial dilutions of the homogenized intestinal caecum tract were plated and the CFU number was enumerated. Statistical significance of protection was obtained using the Mann Whitney test. (B) SslE_IHE3034_ prevents the spread of the UPEC strain 536 into the kidneys and spleen in an ascending model of urinary tract infection. Thirty mice were immunized intranasally with 10 µg cholera toxin (CT) alone or with 100 µg of SslE_IHE3034_ at a 10∶1 ratio of antigen:CT (day 1). After two boosts of 25 µg antigen (10∶1 ratio of antigen to CT) or CT alone (day 7 and 14), mice were transurethrally challenged with 10^8^ CFU of strain 536 at day 21. After 48 h, bladder, kidneys and spleen were harvested and homogenized. Bacteria in urine and in the tissue homogenates were enumerated by plating serial dilutions. Symbols represent CFU/g tissue or CFU/ml urine of individual mice, and bars indicate median values. P values were determined using the nonparametric Mann-Whitney significance test. (C) SslE_IHE3034_ protects against the SEPEC strain IN1S in a sepsis mouse model. CD1 out-bred mice were immunized by subcutaneous injections at day 1, 21, and 35 with 20 µg of recombinant SslE_IHE3034_ formulated with alum or alum alone. Immunized animals were challenged at day 49 with a sublethal dose of heterologous strain IN1S and survival was monitored for up to 4 days. The results are indicated as percentage of survival out of a total number of 40 mice. P values were determined using the nonparametric Mann-Whitney significance test.

Cross-protective efficacy was evaluated by immunizing 30 mice intranasally with the recombinant variant I SslE and challenging them with the ETEC strain GL53 (expressing variant II SslE). Following immunizations with 30 µg of recombinant SslE_IHE3034_ at days 1, 21 and 35, mice were infected by oral gavage with 5×10^7^ CFU of GL53 at day 49. Intestinal caecum tracts were collected at day 51, serial dilutions of the homogenized tissues were plated and the CFU numbers were enumerated. A statistically significant reduction (2.5 Log) in the mean value of GL53 bacterial counts in the caecum was observed in mice immunized with the SslE_IHE3034_ antigen versus those treated with saline (Fig. 8A). Anti-SslE responses in protected mice consisted of antibodies belonging to both IgG and IgA isotypes (Fig. S2C and D).

To further support the observation that SslE_IHE3034_ (variant I) induces heterologous protection, we considered two alternative models: a murine model of ascending UTI and a murine sepsis model. In the UTI model, 30 mice were intranasally inoculated with either cholera toxin (CT) alone (as an adjuvant) or an SslE_IHE3034_-CT mixture. Following three immunizations (days 0, 7, 14), animals were transurethrally challenged on day 21 with the UPEC strain 536 (expressing SslE variant II) and protection was assessed at 48 h post infection by determining the CFUs in the urine, bladder, kidneys and spleen. SslE_IHE3034_ immunization led to a significant reduction in median CFU/g (P = 0.0394) in the kidneys and a more evident protection in the spleen with a 2.0 Log reduction in median CFU/g (P = 0.0006) (Fig. 8B). In the sepsis model, systemic *E. coli* infection was performed as recently reported [10]. Active immunization with SslE_IHE3034_ followed by challenge with the SEPEC strain IN1S (expressing SslE variant II) provided significant protection from mortality (60% survival, P<0.0001) (Fig. 8C).

## Discussion

*E. coli* is a well-adapted human pathogen which uses the gut as a preferential niche and, as for other intestinal microorganisms, it persists in this region due to its ability to exploit a number of metabolic substrates and to stay in the outer mucus layer where commensal bacteria normally reside. Recent studies [30]–[33], including those reported by our group [34], [35], have postulated that this microorganism has adapted to the human body by developing a sophisticated network of virulence and colonization factors. Among these adhesins, iron-uptake systems and IgA binding proteins may allow *E. coli* to out-compete the many species occupying an overcrowded environment such as the intestine. In this scenario, our finding that SslE contributes to *E. coli* mucinase activity suggests the involvement of this antigen in landscaping the *E. coli* territory allowing the establishment of a long lasting colonization. Indeed, shaping of the intestinal microbial community by the mucosa does not only depend on goblet cells secreting antimicrobial proteins, but also on a number of metabolic substrates vital to mucus-degrading bacteria [36]–[38]. In our study, the diminished capacity of the *sslE* mutant strain to translocate through a mucin-rich matrix *in vitro* suggests that SslE activity may facilitate bacterial penetration of the mucosal surface, including the inner mucus layer, to reach the underlying host epithelium. Although these data do not exclude that the catabolism of such glycoproteins may also contribute to an increased fitness of *E. coli* in the outer mucus layer, the pathogenic strains that are armed with immune evasion virulence factors may use SslE as a spearhead to penetrate the sterile inner mucus layer so as to intimately adhere to the epithelial cells of the host.

The core motif, HEXXH, present in SslE is conserved in all families of the Clan of peptidase named MA (M for metallo) although it might also be present by chance in proteins with no peptidase activity [39], [40]. Using the full putative metalloprotease domain of the ExPEC variant of SslE (residues: 1082–1382) to search the Pfam-A protein families database, we confirmed that the entire top 100 hits (E-value<8e-35) were M60-like domains (Pfam ID: PF13402). This domain is exclusively present in a recently characterized zinc metallopeptidase sub-family that possesses mucinase activity [19]. The multiple sequence alignment of the best hits showed the extended motif of the M60-like domain (Supporting information Fig. S4). These hits were mainly bacterial proteins from Gamma proteobacteria, and they have comparable sequence lengths to ExPEC SslE (∼1460–1520 a.a.). Interestingly, the majority of these proteins were predicted to be outer membrane lipoproteins that are N-terminally anchored to the outer membrane, which implies that these mucinases are dedicated to digestion of extracellular host glycoproteins. However, although our data support the hypothesis for the contribution of SslE to *E. coli* colonization by a mechanism likely to involve mucin degradation, we were not able to obtain direct evidence for such an enzymatic activity. Indeed, we observed that recombinant SslE binds to Zinc, but is unable to cleave a number of putative metalloprotease-target molecules including gelatin, casein, fibrinogen, and different collagens (data not shown). However, since bacterial metalloprotease activities are known to depend on different parameters (such as pH, temperature, salt concentration, etc.) [41], [42], further screenings for appropriate *in vitro* conditions will be required.

The large antigenic and genetic variability of pathogenic *E. coli* species has been a major obstacle to the development of a broadly protective vaccine. Indeed, the difficulty in predicting vaccine coverage and the lack of a correlate of protection, has led to numerous promising pre-clinical data not being confirmed by human studies [43]–[47]. By comparing the genome of an ExPEC strain causing neonatal meningitis to those of other ExPEC and nonpathogenic strains, we have recently proposed a number of well conserved protective antigens. Among them the most promising candidate was SslE, which due to its conservation in both intestinal and extraintestinal strains was proposed as a universal vaccine candidate. The anti-mucinase activity exerted by anti-SslE polyclonal antibodies *in vitro*, corroborated by a reduced colonization of caecum in mice immunized with recombinant SslE, further support the hypothesis that the impairment of mucin cleavage may account for the mechanisms of protection from *E. coli* infections in both the mucosal tissues of the gut and the urinary tract [48], [49]. In addition, antibodies generated against SslE variant I showed cross-functional properties versus strains expressing variant II. Since polyclonal antibodies raised against full-length SslE are able to cross-inhibit antigen functional activity, we hypothesized that they may target conserved domains of SslE potentially involved in the metalloprotease activity. However, only a few strains were tested and further studies using a larger panel of clinically relevant strains would be needed to confirm such an assumption.

In conclusion, the contribution of SslE to *E. coli* mucinolytic activity *in vitro*, and SslE mediated protection against intestinal and urinary tract colonization *in vivo*, indicate the importance of SslE as a novel colonization factor and a valid target for intervention strategies against disease caused by this important human pathogen.

## Materials and Methods

### Ethics statement

Animal studies regarding intestinal colonization and sepsis models were carried out in compliance with current Italian legislation on the care and use of animals in experimentation (Legislative Decree 116/92) and with the Novartis Animal Welfare Policy and Standards. Protocols were approved by the Italian Ministry of Health (authorization 236/2010-B) and by the local Novartis Vaccines and Diagnostics Animal Welfare Body (authorization AEC 201010). Animal studies for urinary tract infection experiments were conducted according to protocol #08999 approved by the University Committee on the Care and Use of Animals at the University of Michigan Medical School. The approved procedures are in compliance with University guidelines, State and Federal regulations, and the standards of the “Guide for the Care and Use of Laboratory Animals”.

### Bacterial strains and culture conditions

Genomic DNA was isolated using the GenElute Bacterial Genomic DNA Kit (Sigma) according to the manufacturer's instructions. ExPEC strain IHE3034 (serotype O18:K1:H7) was isolated in Finland in 1976 from a case of human neonatal meningitis [50]. Strains were cultured in Luria-Bertani (LB) broth at 37°C with agitation and aeration. *E. coli* DH5α-T1R (Invitrogen) was used for cloning purposes and *E. coli* BL21(DE3) (Invitrogen) was used for expression of His-tagged fusion proteins. The clones carrying a specific antibiotic resistance cassette were grown in the presence of kanamycin (50 µg/ml) or ampicillin (100 µg/ml).

### Construction of *sslE* deletion mutant and complemented strains

The isogenic *sslE* knockout mutant strain was constructed by replacement of the entire gene by an antibiotic resistance cassette. The upstream and the downstream regions of the *sslE* gene were amplified by PCR with the primers 1–2 and 3–4 (Table S3), using IHE3034 chromosomal DNA as template, and cloned into the pBluescriptKS (Stratagene). The kanamycin resistance cassette was inserted between the two flanking regions in the plasmid. The resulting plasmid was used to electroporate the target strain. Single transformants were confirmed by PCR and Western blotting. Complemented strains were obtained by transformation of the *sslE* mutant with *sslE*_WT and *sslE*_mut recombinant plasmids, carrying the *sslE* wild-type gene or the gene mutated in the putative metallopeptidase motif. For amplification of the *sslE* gene, *E. coli* IHE3034 genomic DNA was used with the primers 5 and 6 (Table S3). The triple mutation (mut) (H1274Y+E1275V+H1278Y) was obtained by two overlapping PCRs performed with primers 7, 8 and 9 (Table S3). Finally, the p*sslE*_WT and p*sslE*_mut constructs were generated carrying the *sslE* predicted promoter region upstream of the *sslE* gene. The two clones harboring these plasmids were produced by a PIPE method [51] that is based on the transformation of HK100 *E. coli* cells with a mix of a vector/insert PCR. The vector PCR was performed using the *sslE*_WT and *sslE*_mut templates with primers 10 and 11 (Table S3), while the insert PCR was obtained with *E. coli* IHE3034 genomic DNA template and primers 12 and 13 (Table S3).

### Confocal staining of pathogenic *E. coli* bacterial cells

*E. coli* strains were grown to exponential phase in LB medium and fixed in PFA 1% for 20 min on a poly-L-lysine-coated slide (Thermo scientific). After a blocking step in PBS+1% BSA, slides were incubated with anti-SslE rabbit serum and then with a donkey anti-rabbit IgG Rhodamine RedX-conjugated antibody (Jackson Immuno-Research Laboratories). IHE3034 bacteria were localized using mouse polyclonal antibodies raised against whole cell IHE3034, and the green fluorescent Alexa Fluor488 goat anti-mouse IgG. The samples were mounted using the Pro-Long Gold antifade reagent containing the blue-fluorescent nuclear counterstain DAPI (Invitrogen). Images were acquired using a 100× oil objective (1.4 n.a.) mounted on a Zeiss LSM710 confocal microscope. In the pictures the signal from SslE was pseudocoloured in green, while the signals from bacteria are shown in red. Z-stacks of images were deconvoluted using Volocity Software (Improvision).

### *In vitro* mucinase activity assays

1. **Amido black assay.** Pathogenic *E. coli* from mid-log culture phase were incubated on LB-agar plates containing 0.5% bovine submaxillary mucin (SIGMA), stained with 0.1% (wt/vol) amido black in 3.5 M acetic acid for 30 min and destained with 1.2 M acetic acid.
2. **Detection of mucinase activity by IVIS.** IHE3034 wild-type, IHE3034Δ*sslE* knockout mutant, IHE3034Δ*sslE*::*sslE_*WT and IHE3034Δ*sslE*::*sslE_*mut were transformed by electroporation with the pGEN-*luxCDABE* plasmid (Amp^R^) expressing luciferase (p*lux* strains). Plasmid stability was assessed in all strains by CFU counting on LB and LB-Amp_100_ plates. A soft gel mucin-based matrix was allowed to polymerize in round 5-ml plates. 5×10^7^ CFU of mid-log *E. coli* p*lux* strains were loaded into wells cut in the middle of the plates and incubated statically at 37°C. Starting inocula were plated to determine the loaded CFU. Pictures were acquired by IVIS 100 time 0 and at 24 h.
3. **Mucin gel degradation assay.** 10^8^ CFU of pathogenic *E. coli* strains from mid-log culture phase were layered on top of 1 ml soft gel-matrix polymerized together with 10% submaxillary gland mucin (SIGMA) in 1 ml syringes and incubated statically for 3 h at 37°C in a vertical position (starting inocula were determined as CFU at time 0). 100 µl fractions were sequentially collected from the bottom of the syringe, diluted and plated. The counts were calculated as % of recovered bacteria in each fraction, sequentially eluted from the column, with respect to the starting inoculum. Data presented are the mean of three independent experiments performed in duplicate. Antibody inhibition of mucin degradation was performed by adding different sera dilutions to the soft gel mucin-based matrix before the polymerization.

### PCR amplification and sequence variability analysis of *sslE* gene

Amplification and sequencing of the *sslE* gene was performed as previously described [10]. Assembly, alignment and comparison of the SslE deduced amino acid sequence was performed with GENEIOUS V6 software (Biomatters. Available from http://www.geneious.com/). In addition to the 96 *sslE* sequences used by Moriel *et al.* [10], 318 *E. coli sslE* sequences were included. The final dataset comprised 414 isolates which comprised EXPEC, InPEC and faecal isolates. Further, sequences relative to unknown *E. coli* pathotypes were extracted from the NCBI database (Table S1). 155 unique SslE protein sequences were selected using GENEIOUS V6 software. The phylogenetic tree was inferred from the alignments by the neighbor-joining distance-based method implemented on MEGA4 [52].

### Cloning, expression and purification of SslE recombinant protein

The *sslE* gene was amplified by PCR from the IHE3034 genomic DNA template, cloned into the pET-21b vector (Novagen) and transformed into DH5α-T1R chemically competent cells for propagation. BL21(DE3) chemically competent cells were used for His-tagged protein expression. The protein was purified by nickel chelating affinity chromatography using a HisTrap HP column (GE Healthcare) followed by anionic exchange chromatography. The purified protein was finally dialyzed in phosphate-buffered saline (PBS) and stored at −20°C.

### *In vivo* monitoring of sslE promoter activity

The P*sslE*-*luxCDABE* plasmid was obtained by replacing the constitutive P*em7* promoter of the pGEN-*luxCDABE* with the *sslE* putative promoter region. To obtain the predicted *sslE* promoter region, a 484-bp fragment was amplified from IHE3034 genomic DNA by PCR using the primers 14 and 15 (Table S3). Chemically competent DH5α cells (Life Technologies) were used for transformation and ampicillin (Amp_100_) was used as a marker of selection. The resulting P*sslE*-*luxCDABE* plasmid was confirmed by sequence analysis and used to transform the ETEC strain GL53 by electroporation, resulting in the GL53-P*sslE-luxCDABE* strain. Ten-week old CD1 female mice (Charles River) were infected intragastrically with 5×10^5^ CFU of either the bioluminescent ETEC GL53-P*sslE-luxCDABE* strain or GL53-P*neg*-*luxCDABE* (promoterless control vector). Imaging of mice anesthetized with isofluorane (4% initially, 1.5% during image acquisition) was performed with an IVIS Spectrum CT Imaging System (Perkin Elmer). Detection of 2D bioluminescent signals was carried out without filters (open), binning 8 and times of acquisition from 1 s to 1 min. 3D images were acquired with six filters (500, 520, 560, 580, 600 and 620 nm), using the same binning and acquisition times and reconstructed by the Living Image software (version 4.3.1).

### Reverse transcriptase-PCR

The GL53 infected caecum was homogenized using gentleMACS Dissociator (MiltenyiBiotec) in 10 ml PBS. After filtration and centrifugation, the pellet was incubated for 5 minutes at room temperature in 3 ml of RNA protect Bacteria Reagent (Qiagen). After cell lysis, total RNA was purified using the RNeasy Mini kit (Qiagen) and an additional DNase treatment was done using the TURBO DNA-free kit (Applied Biosystem), according to the manufacturer's protocols. Purity of RNA was assessed by electrophoresis on agarose gels. Reverse transcription and amplification of an *sslE* fragment with the primers 16 and 17 (Table S3) from RNA were performed using the SuperScript III One-Step RT-PCR System with Platinum Taq DNA Polymerase kit (Invitrogen).

### Mouse model of intestinal colonization

Five-week old CD1 mice were intranasally immunized with 30 µg of SslE antigen at days 1, 21 and 35. Saline was used as a negative control. Fourteen days after the last immunization mice were streptomycin-treated (for 2 days) to eradicate the resident flora and then they were infected by oral gavage with 5×10^7^ CFU/400 µl of strain GL53/Amp^r^. Forty-eight hours after challenge, mice were euthanized and the intestinal caecum tract was recovered and homogenized. Serial dilutions of the suspension were plated on LB/Amp_100_ plates and the CFU were enumerated. Statistical significance of protection was determined using the Mann Whitney test.

### Urinary tract infection model

Female CBA/J mice, 6 to 8 weeks old, were transurethrally inoculated as previously described [53]. Purified antigen was mixed with cholera toxin (CT) (Sigma) at a ratio of 10∶1. The vaccine was administered intranasally in a total volume of 20 µl/animal (10 µl/nostril). Animals received a primary dose on day 0 of 100 µg antigen (containing 10 µg CT) or 10 µg CT alone. Two boosts of 25 µg antigen (mixed with 2.5 µg CT) or 2.5 µg CT alone were given on days 7 and 14, and mice were challenged on day 21. *E. coli* 536 suspensions in phosphate-buffered saline (PBS) (50 µl/mouse) were delivered transurethrally using a sterile 0.28-mm-inner-diameter polyethylene catheter connected to an infusion pump (Harvard Apparatus), with a total inoculum of 10^8^ CFU/mouse. For determination of CFU, organs were aseptically removed from euthanized animals at 48 h post inoculation and homogenized in PBS with a GLH homogenizer (Omni International). Bacteria in tissue homogenates were enumerated by being plated on LB agar containing 0.5 g/liter NaCl using an Autoplate 4000 spiral plater (Spiral Biotech), and CFU were determined using a QCount automated plate counter (Spiral Biotech). Blood was collected as necessary from anesthetized mice by an infraorbital bleed using 1.1- to 1.2-mm Micro-Hematocrit capillary tubes (Fisher), and serum was separated using Microtainer serum separator tubes (Becton Dickinson). The animals were ≤15 weeks old at the conclusion of all experiments.

### Sepsis mouse model

CD1 outbred mice were immunized by subcutaneous injections at day 1, 21, and 35 with 20 µg of recombinant SslE_IHE3034_ formulated with alum or alum alone. Immunized animals were challenged at day 49 with a sublethal dose of a heterologous strain and survival was monitored for up to 4 days. The results are indicated as the percentage of survival from a total number of 40 mice. P values were determined using the nonparametric Mann-Whitney significance test.

### Statistical analysis

Mean values, standard deviation values, and the P values associated to two-tailed unpaired Student's t test were calculated using the Microsoft Excel application. A P value<0.05 was considered statistically significant.

## Supporting Information

Figure S1 Polysialic acid capsule interferes with SslE detection on *E. coli* K1 IHE3034. (A) Flow cytometry detection of K1 capsule on wild-type strain IHE3034 (left panel) and acapsulated IHE3034Δ*kps* strains (right panel) by anti-capsule monoclonal antibody SEAM12 (blue lanes). Serum from animals immunized with PBS was negative control (red). (B) SslE surface detection on wild-type IHE3034 (left panel) and its derivative lacking the capsule IHE3034Δ*kps* (right panel) by anti-SslE immune sera (blue lines) compared to the PBS negative control (red). (C) Titration of binding by an anti-SslE rabbit serum on both IHE3034Δ*kps* acapsulated (red) and IHE3034Δ*kps*Δ*sslE* (blue) strains. (D) Confocal microscopy images of SslE surface localization on IHE3034Δ*kps* and (E) IHE3034Δ*kps*Δ*sslE*. Bacteria were visualized with both DAPI (DNA marker, blue) and FM4–64 Dye (membrane marker, red). SslE was detected using the anti-SslE rabbit serum and a fluorescent secondary antibody (green). Merged images are also displayed. Bars: 2 µm.
(TIF)

Figure S2 IgG and IgA antibody response following SslE immunization. Immunoglobulin levels were quantified by ELISA. Briefly, 100 ng/well of purified SslE was incubated with serial dilution of sera for 2 h at 37°C. Following detection with Alkaline Phosphatase (AP) conjugated secondary antibody, OD_405_ values were plotted in the titration curves. (A) IgG and (B) IgA response derived from serum of immunized rabbit (circles) compared to negative control (square). (C) IgG and (D) IgA response in pool of sera derived from immunized mice (circles) compared to the negative control (square). Each point represents the means ± standard deviations.
(TIF)

Figure S3 Polyclonal antibodies against the truncated C-SslE impair *E. coli* translocation through a mucin matrix. (A) Schematic representation of the C-SslE truncated form lacking the Zn-metalloprotease domain compared to the full-length SslE protein. (B) Inhibition of wild-type IHE3034 translocation through a mucin-gel matrix by anti-C-SslE antibodies compared to negative controls.
(TIF)

Figure S4 Comparison of the SslE core motif with other M60-like members. The figure reports a multiple sequence alignment of the SslE core motif of the zinc metallopeptidase M60-like domain versus the best hits that were found when searching the Pfam-A database. The extended core motif is shown by a dotted square and the conserved residues of the core motif are indicated with an asterisk. The species names are followed by the Uniprot accession codes in brackets.
(TIF)

Table S1 List of strains used for global SslE amino acid sequence alignment.
(PDF)

Table S2 List of SslE unique sequences.
(PDF)

Table S3 List of primers used in the study.
(PDF)

Methods S1 Detailed description of the experimental procedures relative to the data reported in Fig. 6, Fig. S1 and Fig. S2.
(DOCX)

Text S1 Polysialic acid capsule interferes with SslE detection on *E.coli* K1 IHE3034. By comparing the SslE-specific signal between IHE3034 WT and the IHE3034Δkps deletion mutant by FACS and confocal imaging analysis, we demonstrated that the K1 capsule clearly interferes with the anti-SslE antibody accessibility and recognition of the protein on the bacterial surface.
(DOCX)

Text S2 IgG and IgA antibody response following SslE immunization. We observed that subcutaneous immunization of rabbit with recombinant SslE generated a high response in terms of IgG, while IgA values were low.
(DOCX)

Text S3 Polyclonal antibodies against the truncated C-SslE impair *E. coli* translocation through a mucin matrix. We postulated that antibodies against the N-terminal portion of the protein lacking the M60-like zinc-metalloprotease motif are still able to impair *E. coli* translocation through the mucin matrix, suggesting an indirect inhibitory activity.
(DOCX)
