# Correction: SslE Elicits Functional Antibodies That Impair *In Vitro* Mucinase Activity and *In Vivo* Colonization by Both Intestinal and Extraintestinal *Escherichia coli* Strains

**DOI:** 10.1371/journal.ppat.1004773

**Published:** 2015-03-25

**Authors:** The PLOS Pathogens Staff

In the Author Contributions section, Kate L. Seib (KLS) should be listed as one of the persons who conceived and designed the experiments.
