# Supplementary figures and images for: SslE Elicits Functional Antibodies That Impair In Vitro Mucinase Activity and In Vivo Colonization by Both Intestinal and Extraintestinal Escherichia coli Strains
Source: PLoS Pathog. 2014 May 8;10(5):e1004124. doi: 10.1371/journal.ppat.1004124 (PMC4014459; doi:10.1371/journal.ppat.1004124)

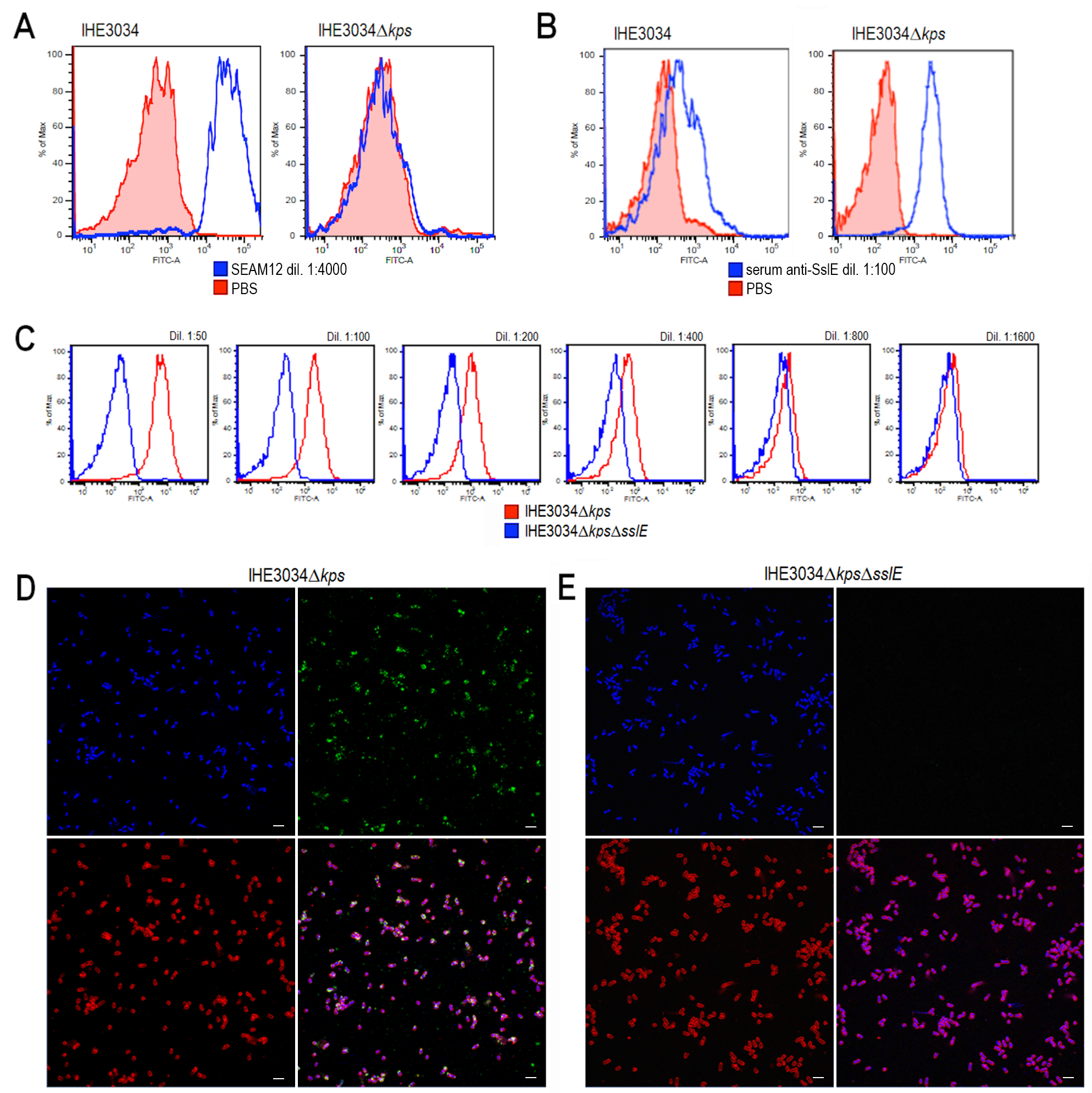

Supplement: Figure S1 — Polysialic acid capsule interferes with SslE detection on E. coli K1 IHE3034. (A) Flow cytometry detection of K1 capsule on wild-type strain IHE3034 (left panel) and acapsulated IHE3034Δkps strains (right panel) by anti-capsule monoclonal antibody SEAM12 (blue lanes). Serum from animals immunized with PBS was negative control (red). (B) SslE surface detection on wild-type IHE3034 (left panel) and its derivative lacking the capsule IHE3034Δkps (right panel) by anti-SslE immune sera (blue lines) compared to the PBS negative control (red). (C) Titration of binding by an anti-SslE rabbit serum on both IHE3034Δkps acapsulated (red) and IHE3034ΔkpsΔsslE (blue) strains. (D) Confocal microscopy images of SslE surface localization on IHE3034Δkps and (E) IHE3034ΔkpsΔsslE. Bacteria were visualized with both DAPI (DNA marker, blue) and FM4–64 Dye (membrane marker, red). SslE was detected using the anti-SslE rabbit serum and a fluorescent secondary antibody (green). Merged images are also displayed. Bars: 2 µm. (TIF) [file ppat.1004124.s001.tif]

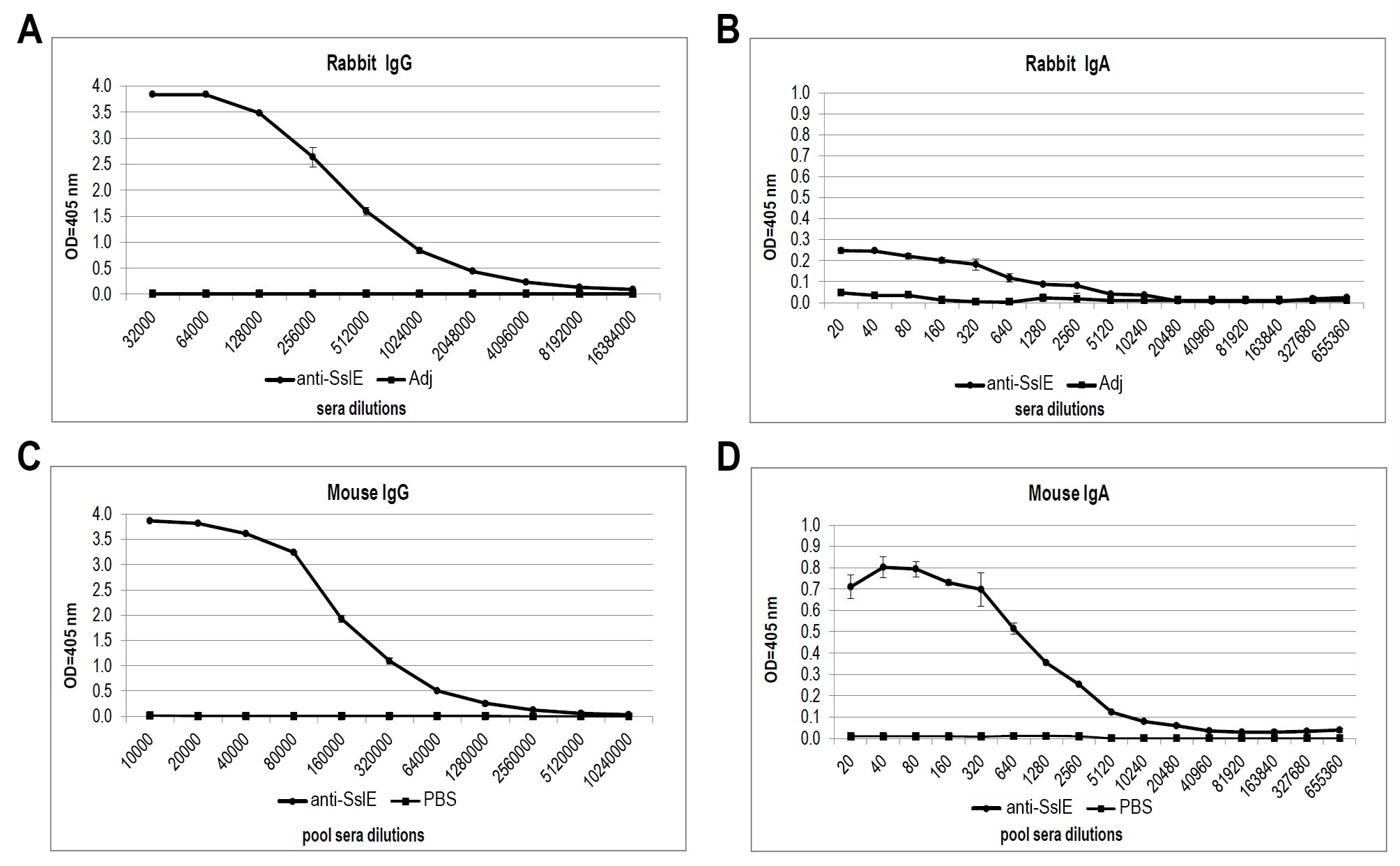

Supplement: Figure S2 — IgG and IgA antibody response following SslE immunization. Immunoglobulin levels were quantified by ELISA. Briefly, 100 ng/well of purified SslE was incubated with serial dilution of sera for 2 h at 37°C. Following detection with Alkaline Phosphatase (AP) conjugated secondary antibody, OD405 values were plotted in the titration curves. (A) IgG and (B) IgA response derived from serum of immunized rabbit (circles) compared to negative control (square). (C) IgG and (D) IgA response in pool of sera derived from immunized mice (circles) compared to the negative control (square). Each point represents the means ± standard deviations. (TIF) [file ppat.1004124.s002.tif]

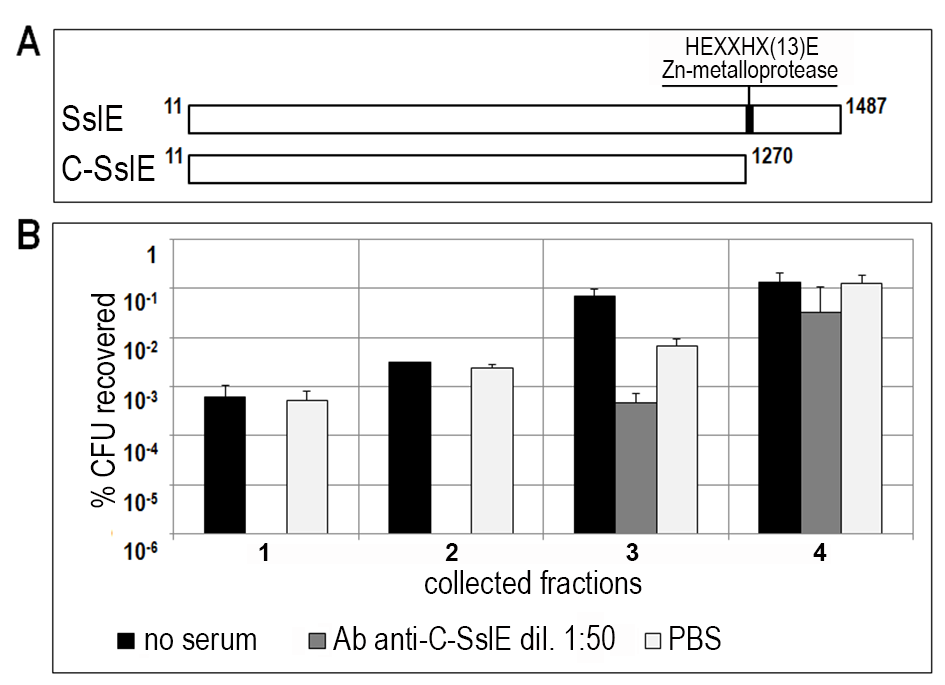

Supplement: Figure S3 — Polyclonal antibodies against the truncated C-SslE impair E. coli translocation through a mucin matrix. (A) Schematic representation of the C-SslE truncated form lacking the Zn-metalloprotease domain compared to the full-length SslE protein. (B) Inhibition of wild-type IHE3034 translocation through a mucin-gel matrix by anti-C-SslE antibodies compared to negative controls. (TIF) [file ppat.1004124.s003.tif]

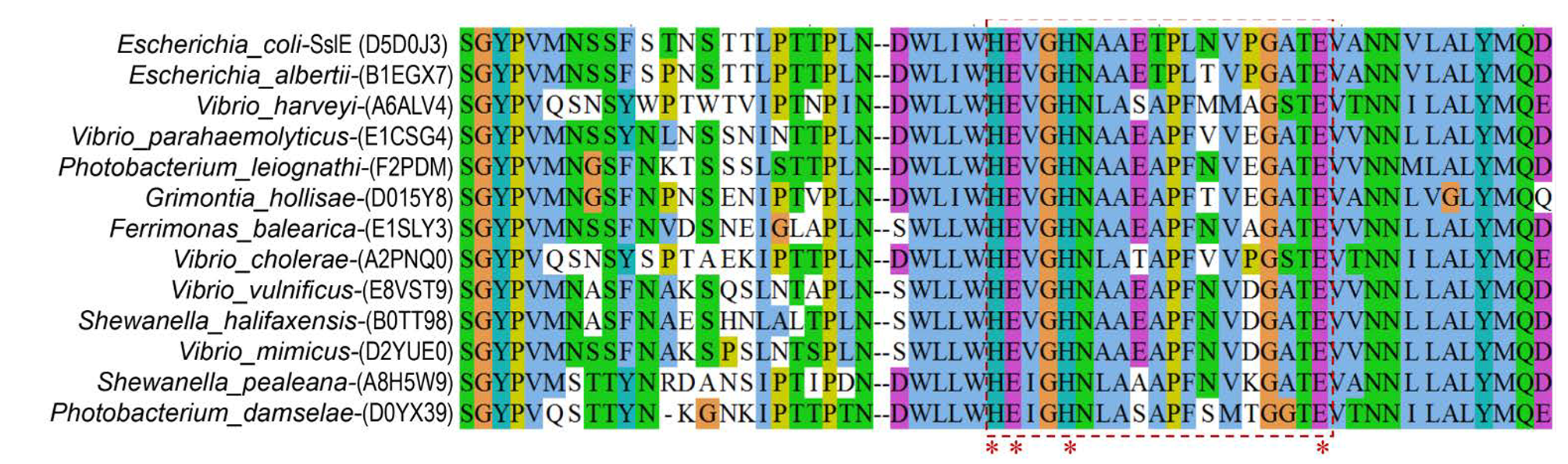

Supplement: Figure S4 — Comparison of the SslE core motif with other M60-like members. The figure reports a multiple sequence alignment of the SslE core motif of the zinc metallopeptidase M60-like domain versus the best hits that were found when searching the Pfam-A database. The extended core motif is shown by a dotted square and the conserved residues of the core motif are indicated with an asterisk. The species names are followed by the Uniprot accession codes in brackets. (TIF) [file ppat.1004124.s004.tif]
