# Supplementary material for: SslE Elicits Functional Antibodies That Impair In Vitro Mucinase Activity and In Vivo Colonization by Both Intestinal and Extraintestinal Escherichia coli Strains
Source: PLoS Pathog. 2014 May 8;10(5):e1004124. doi: 10.1371/journal.ppat.1004124 (PMC4014459; doi:10.1371/journal.ppat.1004124)
Supplement: Table S1 — List of strains used for global SslE amino acid sequence alignment. (PDF) [file ppat.1004124.s005.pdf]

Table S1. Strains used for global SslE amino acid sequence alignment

Strain designation	Main variant type	Sub-variant type	Pathotype	Reference
1939/93	1	1	SePEC	Moriel DG *et al.*, PNAS 2010
4*/S	1	1	ExPEC	Moriel DG *et al.*, PNAS 2010
413/89-1	1	1	STEC	This Study
4549/93	1	1	SePEC	Moriel DG *et al.*, PNAS 2010
ACAC01000020_*Escherichia sp.* 3_2_53FAA	1	1	Not available	This Study
ADTW01000082_*E. coli* MS 110-3	1	1	Not available	This Study
AEFI01000011_*E. coli* H252	1	1	Not available	This Study
AEXH01000054_*E. coli* H397	1	1	Not available	This Study
AEYT01000039_*E. coli* cloneA_i1	1	1	Not available	This Study
AJWU01000192_*E. coli* HM605	1	1	Not available	This Study
APEC01	1	1	APEC	Moriel DG *et al.*, PNAS 2010
B10363	1	1	NMEC	Moriel DG *et al.*, PNAS 2010
B13155	1	1	NMEC	Moriel DG *et al.*, PNAS 2010
B616	1	1	NMEC	Moriel DG *et al.*, PNAS 2010
CADZ01000048_*E. coli* HM605	1	1	Not available	This Study
F18	1	1	Faecal (NP)	This Study
IHE3034	1	1	NMEC	Moriel DG *et al.*, PNAS 2010
IHE3036	1	1	NMEC	Moriel DG *et al.*, PNAS 2010
IHE3080	1	1	NMEC	Moriel DG *et al.*, PNAS 2010
IMT15000	1	1	UPEC	Moriel DG *et al.*, PNAS 2010
IMT15146	1	1	Non-pathogenic	Moriel DG *et al.*, PNAS 2010
IMT2113	1	1	APEC	Moriel DG *et al.*, PNAS 2010
IMT2470	1	1	APEC	Moriel DG *et al.*, PNAS 2010
IMT5124	1	1	APEC	Moriel DG *et al.*, PNAS 2010
IMT5155	1	1	APEC	Moriel DG *et al.*, PNAS 2010
IMT5214	1	1	APEC	Moriel DG *et al.*, PNAS 2010
IMT5215	1	1	APEC	Moriel DG *et al.*, PNAS 2010
IMT9884	1	1	UPEC	Moriel DG *et al.*, PNAS 2010
RS218	1	1	NMEC	Moriel DG *et al.*, PNAS 2010
RS226	1	1	Faecal (NP)	This Study
RW2297	1	1	STEC	This Study
S88	1	1	NMEC	Moriel DG *et al.*, PNAS 2010
U3454	1	1	UPEC	Moriel DG *et al.*, PNAS 2010
UEL31	1	1	APEC	Moriel DG *et al.*, PNAS 2010
UM146	1	1	UPEC	This Study
UTI89	1	1	UPEC	Moriel DG *et al.*, PNAS 2010
IHIT0608	1	11	EHEC	This Study
IMT10740	1	11	Non-pathogenic	Moriel DG *et al.*, PNAS 2010
IMT2283	1	11	APEC	Moriel DG *et al.*, PNAS 2010
IMT8897	1	11	APEC	Moriel DG *et al.*, PNAS 2010
NZ_AEFA01000024_*E. coli* NC101	1	11	Not available	This Study
St5119	1	11	SePEC	Moriel DG *et al.*, PNAS 2010
UR16S	1	11	UPEC	Moriel DG *et al.*, PNAS 2010
269/93	1	17	SePEC	Moriel DG *et al.*, PNAS 2010
ADUF01000307_*E. coli* MS 60-1	1	17	Not available	This Study
F11	1	17	UPEC	Moriel DG *et al.*, PNAS 2010
IMT14993	1	17	UPEC	Moriel DG *et al.*, PNAS 2010
IMT15019	1	17	UPEC	Moriel DG *et al.*, PNAS 2010
IMT5112	1	17	APEC	Moriel DG *et al.*, PNAS 2010
108*/R	1	26	ExPEC	Moriel DG *et al.*, PNAS 2010
70*/R	1	26	ExPEC	Moriel DG *et al.*, PNAS 2010
72*/S	1	26	ExPEC	Moriel DG *et al.*, PNAS 2010
IC14	1	27	EPEC	This Study

IC15	1	27	EPEC	This Study
IC16	1	27	EPEC	This Study
BK658	1	37	NMEC	Moriel DG *et al.*, PNAS 2010
IMT9286	1	37	UPEC	Moriel DG *et al.*, PNAS 2010
IMT9650	1	44	UPEC	Moriel DG *et al.*, PNAS 2010
IMT9713	1	44	APEC	Moriel DG *et al.*, PNAS 2010
79*/R	1	47	Non-pathogenic	This Study
ADUG01000366_*E. coli* MS 57-2	1	47	Not available	This Study
LF82	1	48	AIEC	This Study
O83:H1 str. NRG 857C	1	48	AIEC	This Study
AEHZ01000030_*E. coli* M863	1	49	Not available	This Study
AEZI02000021_*E. coli* 1.2741	1	49	Not available	This Study
AGIA01000038_*E. coli* W26 1.ECW26.1_38	1	50	Not available	This Study
UR7S	2	50	UPEC	Moriel DG *et al.*, PNAS 2010
AMFM01000019_*E. coli* EC302/04	1	51	Not available	This Study
UR36S	1	51	UPEC	Moriel DG *et al.*, PNAS 2010
ADAX01000147_*E. coli* TA206	1	65	Not available	This Study
ADUA01000234_*E. coli* MS 16-3	1	67	Not available	This Study
AEFJ01000016_*E. coli* H263	1	69	Not available	This Study
AEHX01000064_*E. coli* H489	1	71	Not available	This Study
AEJW01000342_*Escherichia sp.* TW09231	1	75	Not available	This Study
AEJX01000130_*Escherichia sp.* TW15838	1	76	Not available	This Study
AEKA01000038_*Escherichia sp*. TW10509	1	78	Not available	This Study
AEXD01000018_*E. coli* STEC_7v	1	84	STEC	This Study
AJFG01000026_*E. coli* SCI-07	1	107	Not available	This Study
AJWO01000049_*E. coli* KD1	1	111	Not available	This Study
ED1a	1	122	Non-pathogenic	This Study
IMT14973	1	134	UPEC	Moriel DG *et al.*, PNAS 2010
IMT15009	1	135	UPEC	Moriel DG *et al.*, PNAS 2010
IMT15150	1	137	Non-pathogenic	Moriel DG *et al.*, PNAS 2010
IMT2121	1	139	APEC	Moriel DG *et al.*, PNAS 2010
NZ_ACDM02000041_*Escherichia sp.* 4_1_40B	1	146	Not available	This Study
NZ_ADUC01000346_*E. coli* MS 200-1	1	151	Not available	This Study
NZ_AEME01000001_*Escherichia sp.* TW09308	1	153	Not available	This Study
55989	2	2	EAEC	This Study
AFOG01000118_*E. coli* O104:H4 str. TY-2482	2	2	Not available	This Study
AFPN02000015_*E. coli* O104:H4 str. H112180280	2	2	Not available	This Study
AFRH01000009_*E. coli* O104:H4 str. C227-11	2	2	Not available	This Study
AFRI01000008_*E. coli* O104:H4 str. C236-11	2	2	Not available	This Study
AFRM01000014_*E. coli* O104:H4 str. 11-3677	2	2	Not available	This Study
AFSO01000022_*E. coli* O104:H4 str. H112180282 CS22	2	2	Not available	This Study
AFUX01000016_*E. coli* O104:H4 str. 11-4404	2	2	Not available	This Study
AFUY01000014_*E. coli* O104:H4 str. 11-4522	2	2	Not available	This Study
AFUZ01000014_*E. coli* O104:H4 str. 11-4623	2	2	Not available	This Study
AFVB01000001_*E. coli* O104:H4 str. 11-4632 C2	2	2	Not available	This Study
AFVC01000015_*E. coli* O104:H4 str. 11-4632 C3	2	2	Not available	This Study
AFVD01000025_*E. coli* O104:H4 str. 11-4632	2	2	Not available	This Study
AFVE01000003_*E. coli* O104:H4 str. 11-4632 C5	2	2	Not available	This Study
AFVR01000009_*E. coli* O104:H4 str. TY-2482	2	2	Not available	This Study
AFWO01000033_*E. coli* O104:H4 str. GOS1	2	2	Not available	This Study
AFWP01000023_*E. coli* O104:H4 str. GOS2	2	2	Not available	This Study
AHZE01000047_*E. coli* O104:H4 str. ON2010	2	2	Not available	This Study
AHZF01000082_*E. coli* O104:H4 str. ON2011	2	2	Not available	This Study
LB226692	2	2	EHAEC	This Study
O104:H4 str. 11-4632 C1	2	2	Not available	This Study
764	2	3	UPEC	Moriel DG *et al.*, PNAS 2010
9855/93	2	3	SePEC	Moriel DG *et al.*, PNAS 2010

ADTX01000372_*E. coli* MS 153-1	2	3	Not available	This Study
E457	2	3	Faecal (NP)	This Study
Ecor64	2	3	UPEC	Moriel DG *et al.*, PNAS 2010
IN16R	2	3	SePEC	Moriel DG *et al.*, PNAS 2010
IN22R	2	3	SePEC	Moriel DG *et al.*, PNAS 2010
IN30R	2	3	SePEC	Moriel DG *et al.*, PNAS 2010
IN31R	2	3	SePEC	Moriel DG *et al.*, PNAS 2010
IN32R	2	3	SePEC	Moriel DG *et al.*, PNAS 2010
IN33R	2	3	SePEC	Moriel DG *et al.*, PNAS 2010
IN36R	2	3	SePEC	Moriel DG *et al.*, PNAS 2010
IN40R	2	3	UPEC	Moriel DG *et al.*, PNAS 2010
IN6R	2	3	SePEC	Moriel DG *et al.*, PNAS 2010
Nissle	2	3	Non-pathogenic	This Study
NZ_ACGN01000126_*E. coli* 83972	2	3	Not available	This Study
NZ_ADTO01000027_*E. coli* MS 45-1	2	3	Not available	This Study
UR14R	2	3	UPEC	Moriel DG *et al.*, PNAS 2010
UR39R	2	3	UPEC	Moriel DG *et al.*, PNAS 2010
UR40R	2	3	UPEC	Moriel DG *et al.*, PNAS 2010
AFAH02000003_*E. coli* B41	2	4	Not available	This Study
AFEA01000015_*E. coli* STEC_S1191	2	4	STEC	This Study
AFVX01000037_*E. coli* XH140A	2	4	Not available	This Study
AFYG01000011_*E. coli* XH001	2	4	Not available	This Study
AGTD01000001_*E. coli* UMNF18	2	4	Not available	This Study
AICK01000008_*E. coli* J53 1.ECJ53.1_8	2	4	Not available	This Study
AIFV01000029_*E. coli* DEC6A	2	4	Not available	This Study
AIFW01000041_*E. coli* DEC6B	2	4	Not available	This Study
BW2952	2	4	Non-pathogenic	This Study
DH10B	2	4	Non-pathogenic	This Study
F41_GL633	2	4	ETEC	This Study
IC17	2	4	EPEC	This Study
IC23	2	4	EPEC	This Study
K99_NAH1043	2	4	ETEC	This Study
MG1655	2	4	Non-pathogenic	This Study
MM294.1	2	4	Non-pathogenic	This Study
NZ_ADUB01000340_*E. coli* MS 175-1	2	4	Not available	This Study
W3110	2	4	Non-pathogenic	This Study
AFJB01000030_*E. coli* DEC2B	2	5	Not available	This Study
AIEV01000046_*E. coli* DEC1A	2	5	Not available	This Study
AIEW01000055_*E. coli* DEC1B	2	5	Not available	This Study
AIEX01000059_*E. coli* DEC1C	2	5	Not available	This Study
AIEY01000055_*E. coli* DEC1D	2	5	Not available	This Study
AIEZ01000029_*E. coli* DEC1E	2	5	Not available	This Study
AIFA01000057_*E. coli* DEC2A	2	5	Not available	This Study
AIFB01000055_*E. coli* DEC2C	2	5	Not available	This Study
AIFC01000033_*E. coli* DEC2D	2	5	Not available	This Study
AIFD01000045_*E. coli* DEC2E	2	5	Not available	This Study
E2348/69	2	5	EPEC	This Study
IC37	2	5	EPEC	This Study
IC39	2	5	EPEC	This Study
IC44	2	5	EPEC	This Study
IC46	2	5	EPEC	This Study
NZ_ADUL01000074_*E. coli* 2362-75	2	5	Not available	This Study
O55_H7	2	5	EPEC	This Study
3970	2	6	ETEC	This Study
89*/S	2	6	Non-pathogenic	Moriel DG *et al.*, PNAS 2010
ADAZ01000127_*E. coli* TA271	2	6	Not available	This Study
ADBB01000134_*E. coli* H591	2	6	Not available	This Study

AEZN01000047_*E. coli* 95.0941	2	6	Not available	This Study
AEZP01000103_*E. coli* 97.0264	2	6	Not available	This Study
AEZW02000001_*E. coli* 96.154	2	6	Not available	This Study
AFAC02000011_*E. coli* 3.3884	2	6	Not available	This Study
AGTH01000234_*E. coli* O113:H21 str. CL-3 EC1623	2	6	Not available	This Study
AHWG01000027_*E. coli* B799	2	6	Not available	This Study
Ecor34	2	6	Non-pathogenic	This Study
IMT10666	2	6	Non-pathogenic	This Study
IMT1939	2	6	APEC	Moriel DG *et al.,* PNAS 2010
NZ_ADWU01000032_*E. coli* MS 119-7	2	6	Not available	This Study
ADUQ01000066_*E. coli* OK1180	2	7	Not available	This Study
AEZU02000102_*E. coli* 4.0522	2	7	Not available	This Study
AEZV02000064_*E. coli* JB1-95	2	7	Not available	This Study
AFDS01000066_*E. coli* 2534-86	2	7	Not available	This Study
AIGF01000039_*E. coli* DEC8A	2	7	Not available	This Study
AIGG01000066_*E. coli* DEC8B	2	7	Not available	This Study
AIGJ01000060_*E. coli* DEC8E	2	7	Not available	This Study
AIGU01000059_*E. coli* DEC10F	2	7	Not available	This Study
AJVU01000556_*E. coli* O111:H8 str. CVM9570 ECO9570	2	7	Not available	This Study
AJVV01000013_*E. coli* O111:H8 str. CVM9574 ECO9574	2	7	Not available	This Study
AKAV01000247_*E. coli* O111:H8 str. CVM9602 ECO9602	2	7	Not available	This Study
AKAW01000035_*E. coli* O111:H8 str. CVM9634 ECO9634_13	2	7	Not available	This Study
O111:H- str. 11128	2	7	EHEC	This Study
AEVZ01000051_*E. coli* M919	2	8	Not available	This Study
AEZS02000010_*E. coli* 3.2608	2	8	Not available	This Study
AEZT02000043_*E. coli* 93.0624	2	8	Not available	This Study
AEZX02000008_*E. coli* 5.0959	2	8	Not available	This Study
AGTJ01000171_*E. coli* O121:H19 str. MT#2 EC1660	2	8	Not available	This Study
AJVR01000250_*E. coli* O103:H2 str. CVM9450 ECO9450	2	8	Not available	This Study
IMT2312	2	8	APEC	Moriel DG *et al.,* PNAS 2010
RL318/96	2	8	EPEC	This Study
RW1374	2	8	EHEC	This Study
A5/10	2	9	STEC	This Study
ADTS01000262_*E. coli* MS 117-3	2	9	Not available	This Study
AIHO01000047_*E. coli* DEC15A	2	9	Not available	This Study
AIHP01000026_*E. coli* DEC15B	2	9	Not available	This Study
AIHQ01000023_*E. coli* DEC15C	2	9	Not available	This Study
AIHR01000024_*E. coli* DEC15D	2	9	Not available	This Study
AIHS01000038_*E. coli* DEC15E	2	9	Not available	This Study
IC1	2	9	EPEC	This Study
SE11	2	9	Non-pathogenic	This Study
10968/93	2	10	SePEC	Moriel DG *et al.,* PNAS 2010
ACXI01000095_*E. coli* FVEC1412	2	10	Not available	This Study
ADAY01000124_*E. coli* TA143	2	10	Not available	This Study
AJWS01000282_*E. coli* 576-1	2	10	Not available	This Study
FVEC1302_*E. coli* FVEC1302	2	10	Not available	This Study
IN1S	2	10	SePEC	Moriel DG *et al.,* PNAS 2010
NZ_ADTJ01000429_*E. coli* MS 198-1 *E_coli* 198-1-1.0	2	10	Not available	This Study
UMNO26	2	10	UPEC	Moriel DG *et al.,* PNAS 2010
AFEB01000047_*E. coli* TX1999	2	12	Not available	This Study
AIGA01000047_*E. coli* DEC7A	2	12	Not available	This Study
AIGC01000017_*E. coli* DEC7C	2	12	Not available	This Study
AIGD01000020_*E. coli* DEC7D	2	12	Not available	This Study
AIGE01000027_*E. coli* DEC7E	2	12	Not available	This Study
AKNH01000052_*E. coli* EPECa12	2	12	EPEC	This Study
K88_GL53	2	12	ETEC	This Study
ADWQ01000035_*E. coli* MS 85-1	2	13	Not available	This Study

IMT15006	2	13	UPEC	Moriel DG *et al.*, PNAS 2010
IMT15020	2	13	UPEC	Moriel DG *et al.*, PNAS 2010
IMT2120	2	13	APEC	Moriel DG *et al.*, PNAS 2010
MGS_104	2	13	Faecal (NP)	This Study
NZ_ADTK01000302_*E. coli* MS 84-1 *E. coli* 84-1-1.0	2	13	Not available	This Study
NZ_ADWT01000020_*E. coli* MS 124-1 E	2	13	Not available	This Study
AFAA02000008_*E. coli* 4.0967	2	14	Not available	This Study
AIGV01000047_*E. coli* DEC11A	2	14	Not available	This Study
AIGW01000036_*E. coli* DEC11B	2	14	Not available	This Study
E22	2	14	EPEC	This Study
IC11	2	14	EPEC	This Study
IC12	2	14	EPEC	This Study
IC13	2	14	EPEC	This Study
E1619	2	15	ETEC	This Study
E1649	2	15	ETEC	This Study
E3007	2	15	ETEC	This Study
E3008	2	15	ETEC	This Study
F911	2	15	SePEC	Moriel DG *et al.*, PNAS 2010
TW10598	2	15	ETEC	This Study
AIHF01000024_*E. coli* DEC13A	2	16	Not available	This Study
AIHG01000024_*E. coli* DEC13B	2	16	Not available	This Study
AIHH01000053_*E. coli* DEC13C	2	16	Not available	This Study
AIHI01000022_*E. coli* DEC13D	2	16	Not available	This Study
AIHJ01000038_*E. coli* DEC13E	2	16	Not available	This Study
E3014	2	16	ETEC	This Study
101-1	2	18	EAEC	This Study
ADUS01000030_*E. coli* RN587/1	2	18	Not available	This Study
AFAF02000002_*E. coli* 3003	2	18	Not available	This Study
AMUL01000155_*E. coli* ARS4.2123	2	18	Not available	This Study
E2593	2	18	ETEC	This Study
AEUB01000046_*E. coli* O55:H7 str. USDA 5905 ECO5905	2	19	Not available	This Study
AIFS01000039_*E. coli* DEC5C	2	19	Not available	This Study
AIFT01000031_*E. coli* DEC5D	2	19	Not available	This Study
AMUG01000378_*E. coli* 5905	2	19	Not available	This Study
IC48	2	19	EPEC	This Study
E3009	2	20	ETEC	This Study
E3020	2	20	ETEC	This Study
E57	2	20	ETEC	This Study
E97	2	20	ETEC	This Study
NZ_ADTM01000140_*E. coli* MS 182-1	2	20	Not available	This Study
ADBC01000136_*E. coli* H299	2	21	Not available	This Study
IMT15014	2	21	UPEC	Moriel DG *et al.*, PNAS 2010
IMT2358	2	21	APEC	Moriel DG *et al.*, PNAS 2010
IMT2490	2	21	APEC	Moriel DG *et al.*, PNAS 2010
AEHU01000029_*E. coli* E482	2	22	Not available	This Study
E1257	2	22	ETEC	This Study
HS	2	22	Non-pathogenic	This Study
NZ_ADUK01000025_*E. coli* 1827-70	2	22	Not available	This Study
IC34	2	23	EPEC	This Study
IC35	2	23	EPEC	This Study
IC36	2	23	EPEC	This Study
IC38	2	23	EPEC	This Study
B7A	2	24	ETEC	This Study
EDL899	2	24	ETEC	This Study
NZ_ADWS01000004_*E. coli* MS 145-7	2	24	Not available	This Study
UR36R	2	24	UPEC	Moriel DG *et al.*, PNAS 2010
AIGL01000051_*E. coli* DEC9B	2	25	Not available	This Study

AIGM01000045_*E. coli* DEC9C	2	25	Not available	This Study
AIGN01000054_*E. coli* DEC9D	2	25	Not available	This Study
IC40	2	25	EPEC	This Study
AEUA01000070_*E. coli* O55:H7 str. 3256-97	2	28	Not available	This Study
AIFR01000046_*E. coli* DEC5B	2	28	Not available	This Study
IC49	2	28	EPEC	This Study
AEZO02000013_*E. coli* 1.2264	2	29	Not available	This Study
E3003	2	29	ETEC	This Study
TW10722	2	29	ETEC	This Study
E1584	2	30	ETEC	This Study
E3006	2	30	ETEC	This Study
TX-1	2	30	ETEC	This Study
IC50	2	31	EPEC	This Study
IC51	2	31	EPEC	This Study
IC52	2	31	EPEC	This Study
AEZQ02000013_*E. coli* 96.0497	2	32	Not available	This Study
AFDQ01000026_*E. coli* STEC_B2F1	2	32	STEC	This Study
AGTI01000009_*E. coli* O91:H21 str. B2F1 EC1624	2	32	Not available	This Study
AIGT01000031_*E. coli* DEC10E	2	33	Not available	This Study
AIHL01000068_*E. coli* DEC14B	2	33	Not available	This Study
AIHN01000056_*E. coli* DEC14D	2	33	Not available	This Study
AHWF01000040_*E. coli* H730	2	34	Not available	This Study
IAI39	2	34	UPEC	Moriel DG *et al.*, PNAS 2010
RS179	2	34	NMEC	Moriel DG *et al.*, PNAS 2010
IMT16101	2	35	Non-pathogenic	Moriel DG *et al.*, PNAS 2010
NZ_ADUE01000153_*E. coli* MS 185-1	1	35	Not available	This Study
UR41S	2	35	UPEC	Moriel DG *et al.*, PNAS 2010
AFPS01000089_*E. coli* O104:H4 str. 01-09591 HUSEC41_3427361-3995069_bb	2	36	Not available	This Study
AFRK01000009_*E. coli* O104:H4 str. 09-7901	2	36	Not available	This Study
AFRL01000011_*E. coli* O104:H4 str. 04-8351	2	36	Not available	This Study
AFEX01000035_*E. coli* STEC_O31	2	38	STEC	This Study
IC22	2	38	EPEC	This Study
IC5	2	39	EPEC	This Study
IC6	2	39	EPEC	This Study
E110019	2	40	EPEC	This Study
NZ_AAJW02000002_*E. coli* E110019	2	40	Not available	This Study
Ecor35	2	41	Non-pathogenic	This Study
RS168	2	41	NMEC	Moriel DG *et al.*, PNAS 2010
IC41	2	42	EPEC	This Study
IC43	2	42	EPEC	This Study
AFET01000002_*E. coli* AA86 53_2	2	43	Not available	This Study
M605_*E. coli* M605	2	43	Not available	This Study
536	2	45	UPEC	Moriel DG *et al.*, PNAS 2010
E642	2	45	Not available	This Study
537/89	2	46	STEC	This Study
AJVQ01000230_*E. coli* O103:H25 str. CVM9340 ECO9340_34	2	46	Not available	This Study
BL21DE3	2	52	Non-pathogenic	This Study
REL606	2	52	Non-pathogenic	This Study
53638	2	53	EIEC	This Study
2656/93	2	54	SePEC	Moriel DG *et al.*, PNAS 2010
2770/93	2	55	SePEC	Moriel DG *et al.*, PNAS 2010
2882/93	2	56	SePEC	Moriel DG *et al.*, PNAS 2010
47*/R	2	57	ExPEC	Moriel DG *et al.*, PNAS 2010
987P_NAH1073	2	58	ETEC	This Study
ACTQ01000026_*E. coli* 4_1_47FAA	2	60	Not available	This Study
ACXE01000093_*E. coli* B088	2	61	Not available	This Study

ACXF01000061_*E. coli* B185	2	62	Not available	This Study
ACXG01000045_*E. coli* B354	2	63	Not available	This Study
ADAW01000125_*E. coli* M718	2	64	Not available	This Study
ADBA01000109_*E. coli* TA280	2	66	Not available	This Study
ADUR01000068_*E. coli* OK1357	2	68	Not available	This Study
AEHS01000005_*E. coli* E1167	2	70	Not available	This Study
AEIA01000002_*Escherichia fergusonii* B253	2	72	Not available	This Study
AEJV01000091_*Escherichia sp.* TW09276	2	74	Not available	This Study
AEMF01000001_*E. albertii* TW11588	2	79	Not available	This Study
AERT01000019_*E. coli* WV_060327	2	80	Not available	This Study
AERU01000008_*E. coli* EC4100B	2	81	Not available	This Study
AEUC01000074_*E. coli* O157:H7 str. LSU-61 ECOSU61	2	82	Not available	This Study
AEVY01000037_*Escherichia fergusonii* ECD227	2	83	Not available	This Study
AEXF01000005_*E. coli* B093	2	85	Not available	This Study
AEXG01000004_*E. coli* E101	2	86	Not available	This Study
AEZJ02000017_*E. coli* 97.0246	2	87	Not available	This Study
AEZK02000026_*E. coli* 5.0588	2	88	Not available	This Study
AEZL02000036_*E. coli* 97.0259	2	89	Not available	This Study
AFAB02000133_*E. coli* 2.3916	2	90	Not available	This Study
AFAG02000002_*E. coli* TW07793	2	91	Not available	This Study
AFAT01000060_*E. coli* PCN033	2	92	Not available	This Study
AFDR01000025_*E. coli* STEC_C165-02	2	93	STEC	This Study
AFDT01000052_*E. coli* 3030-1	2	94	Not available	This Study
AFDU01000023_*E. coli* STEC_94C	2	95	STEC	This Study
AFDV01000052_*E. coli* STEC_DG131-3	2	96	STEC	This Study
AFDY01000038_*E. coli* STEC_H.1.8	2	97	STEC	This Study
AFDZ01000021_*E. coli* STEC_MHI813	2	98	STEC	This Study
AFQH01000043_*E. coli* H494	2	99	Not available	This Study
AFQI01000003_*E. coli* TA124	2	100	Not available	This Study
AGSG01000200_*E. coli* O103:H25 str. NIPH-11060424	2	101	Not available	This Study
AIFQ01000025_*E. coli* DEC5A	2	102	Not available	This Study
AIFU01000020_*E. coli* DEC5E	2	103	Not available	This Study
AIGO01000051_*E. coli* DEC9E	2	104	Not available	This Study
AIHK01000025_*E. coli* DEC14A	2	105	Not available	This Study
AIHM01000060_*E. coli* DEC14C	2	106	Not available	This Study
AJLU01000015_*E. coli* NCCP15657	2	108	Not available	This Study
AJMB01000011_*E. coli* NCCP15647	2	109	Not available	This Study
AJPQ01000002_*E. coli* AI27 1.ECAI27.1_2	2	110	Not available	This Study
AJWP01000185_*E. coli* KD2	2	112	Not available	This Study
AJWR01000021_*E. coli* 541-1	2	113	Not available	This Study
AJWV01000019_*E. coli* CUMT8	2	114	Not available	This Study
AMSK01000003_*E. coli* AD30 1.ECAD30.1_3	2	115	Not available	This Study
AMUP01000193_*E. coli* 07798 E07798.contig.187_2	2	116	Not available	This Study
AMVJ01000109_*E. coli* 0.1288 E01288	2	117	Not available	This Study
CAFL01000093_*E. coli* O25b:H4-ST131 str. EC958	2	118	Not available	This Study
E1593	2	119	ETEC	This Study
E24377A	2	120	ETEC	This Study
E351	2	121	Not available	Moriel DG *et al.*, PNAS 2010
EDL1943	2	123	ETEC	This Study
F645	2	124	SePEC	Moriel DG *et al.*, PNAS 2010
H10407	2	125	ETEC	This Study
IAI1	2	126	Non-pathogenic	Moriel DG *et al.*, PNAS 2010
IC18	2	127	EPEC	This Study
IC19	2	128	EPEC	This Study
IC21	2	129	EPEC	This Study
IC28	2	130	EPEC	This Study
IC33	2	131	EPEC	This Study

IC4	2	132	EPEC	This Study
IC47	2	133	EPEC	This Study
IMT15010	2	136	UPEC	Moriel DG *et al.,* PNAS 2010
IMT2111	2	138	APEC	Moriel DG *et al.,* PNAS 2010
IMT8103	2	140	UPEC	Moriel DG *et al.,* PNAS 2010
IMT9087	2	141	UPEC	Moriel DG *et al.,* PNAS 2010
MGS_124	2	142	Faecal (NP)	This Study
MGS_73	2	143	Faecal (NP)	This Study
MGS_89	2	144	Faecal (NP)	This Study
NZ_ACID02000116_*Escherichia sp.* 1_1_43	2	147	Not available	This Study
NZ_ADTQ01000274_*E. coli* MS 187-1	2	148	Not available	This Study
NZ_ADTY01000057_*E. coli* MS 78-1	2	149	Not available	This Study
NZ_ADTZ01000399_*E. coli* MS 116-1	2	150	Not available	This Study
NZ_ADUD01000094_*E. coli* MS 196-1	2	152	Not available	This Study
O2	2	154	Not available	Moriel DG *et al.,* PNAS 2010
O42	2	155	EAEC	This Study
O78	2	156	Not available	Moriel DG *et al.,* PNAS 2010
SE15	2	157	Non-pathogenic	This Study
SMS3_5	2	158	Non-pathogenic	This Study
TW10828	2	159	ETEC	This Study
TW14425	2	160	ETEC	This Study
U5070	2	161	UPEC	Moriel DG *et al.,* PNAS 2010
W	2	162	Non-pathogenic	This Study
AcfD *Vibrio cholerae*	none	59	none	This Study
AEJU01000035_*E. albertii* TW08933	none	73	Not available	This Study
AEJY01000042_*E. albertii* TW15818	none	77	Not available	This Study
NZ_ABKX01000001_*E. albertii* TW07627	none	145	Not available	This Study
