# Supplementary material for: SslE Elicits Functional Antibodies That Impair In Vitro Mucinase Activity and In Vivo Colonization by Both Intestinal and Extraintestinal Escherichia coli Strains
Source: PLoS Pathog. 2014 May 8;10(5):e1004124. doi: 10.1371/journal.ppat.1004124 (PMC4014459; doi:10.1371/journal.ppat.1004124)
Supplement: Table S2 — List of SslE unique sequences. (PDF) [file ppat.1004124.s006.pdf]

Table S2. SslE unique sequences

Strain designation	Main variant type	Sub-variant type	No. of strains with SslE identical sequence
IHE3034	1	1	36
LB226692	2	2	21
9855/93	2	3	20
AGTD01000001_*Escherichia coli* UMNF18	2	4	18
NZ_ADUL01000074_*Escherichia coli* 2362-75	2	5	17
AGTH01000234_*Escherichia coli* O113H21 str. CL-3 EC1623	2	6	14
ADUQ01000066_*Escherichia coli* OK1180	2	7	13
AJVR01000250_*Escherichia coli* O103H2 str. CVM9450 ECO9450	2	8	9
AIHP01000026_*Escherichia coli* DEC15B	2	9	9
IN1S	2	10	8
UR16S	1	11	7
K88_GL53	2	12	7
IMT15006	2	13	7
IC11	2	14	7
TW10598	2	15	6
AIHJ01000038_*Escherichia coli* DEC13E	2	16	6
IMT5112	1	17	6
ADUS01000030_*Escherichia coli* RN587/1	2	18	5
AEUB01000046_*Escherichia coli* O55H7 str. USDA 5905 ECO5905	2	19	5
E57	2	20	5
IMT2358	2	21	4
HS	2	22	4
IC35	2	23	4
B7A	2	24	4
AIGM01000045_*Escherichia coli* DEC9C	2	25	4
70*/R	1	26	3
IC15	1	27	3
IC49	2	28	3
AEZO02000013_*Escherichia coli* 1.2264	2	29	3
E3006	2	30	3
IC50	2	31	3
AGTI01000009_*Escherichia coli* O91H21 str. B2F1 EC1624	2	32	3
AIHN01000056_*Escherichia coli* DEC14D	2	33	3
AHWF01000040_*Escherichia coli* H730	2	34	3
NZ_ADUE01000153_*Escherichia coli* MS 185-1	1	35	3
AFRL01000011_*Escherichia coli* O104H4 str. 04-8351	2	36	3
IMT9286	1	37	2
AFEX01000035_*Escherichia coli* STEC_O31	2	38	2
IC6	2	39	2
E110019	2	40	2
RS168	2	41	2
IC43	2	42	2
AFET01000002_*Escherichia coli* AA86 53_2	2	43	2
IMT9650	1	44	2
536	2	45	2
537/89	2	46	2
ADUG01000366_*Escherichia coli* MS 57-2	1	47	2
LF82	1	48	2
AEZI02000021_*Escherichia coli* 1.2741	1	49	2
UR7S	2	50	2
UR36S	1	51	2
REL606	2	52	2
53638	2	53	1

2656/93	2	54	1
2770/93	2	55	1
2882/93	2	56	1
47*/R	2	57	1
987P_NAH1073	2	58	1
AcfD *Vibrio cholerae*	none	59	1
ACTQ01000026_*Escherichia coli* 4_1_47FAA	2	60	1
ACXE01000093_*Escherichia coli* B088	2	61	1
ACXF01000061_*Escherichia coli* B185	2	62	1
ACXG01000045_*Escherichia coli* B354	2	63	1
ADAW01000125_*Escherichia coli* M718	2	64	1
ADAX01000147_*Escherichia coli* TA206	1	65	1
ADBA01000109_*Escherichia coli* TA280	2	66	1
ADUA01000234_*Escherichia coli* MS 16-3	1	67	1
ADUR01000068_*Escherichia coli* OK1357	2	68	1
AEFJ01000016_*Escherichia coli* H263	1	69	1
AEHS01000005_*Escherichia coli* E1167	2	70	1
AEHX01000064_*Escherichia coli* H489	1	71	1
AEIA01000002_*Escherichia fergusonii* B253	2	72	1
AEJU01000035_*Escherichia albertii* TW08933	none	73	1
AEJV01000091_*Escherichia sp.* TW09276	2	74	1
AEJW01000342_*Escherichia sp.* TW09231	1	75	1
AEJX01000130_*Escherichia sp.* TW15838	1	76	1
AEJY01000042_*Escherichia albertii* TW15818	none	77	1
AEKA01000038_*Escherichia sp.* TW10509	1	78	1
AEMF01000001_*Escherichia albertii* TW11588	2	79	1
AERT01000019_*Escherichia coli* WV_060327	2	80	1
AERU01000008_*Escherichia coli* EC4100B	2	81	1
AEUC01000074_*Escherichia coli* O157H7 str. LSU-61 ECOSU61	2	82	1
AEVY01000037_*Escherichia fergusonii* ECD227	2	83	1
AEXD01000018_*Escherichia coli* STEC_7v	1	84	1
AEXF01000005_*Escherichia coli* B093	2	85	1
AEXG01000004_*Escherichia coli* E101	2	86	1
AEZJ02000017_*Escherichia coli* 97.0246	2	87	1
AEZK02000026_*Escherichia coli* 5.0588	2	88	1
AEZL02000036_*Escherichia coli* 97.0259	2	89	1
AFAB02000133_*Escherichia coli* 2.3916	2	90	1
AFAG02000002_*Escherichia coli* TW07793	2	91	1
AFAT01000060_*Escherichia coli* PCN033	2	92	1
AFDR01000025_*Escherichia coli* STEC_C165-02	2	93	1
AFDT01000052_*Escherichia coli* 3030-1	2	94	1
AFDU01000023_*Escherichia coli* STEC_94C	2	95	1
AFDV01000052_*Escherichia coli* STEC_DG131-3	2	96	1
AFDY01000038_*Escherichia coli* STEC_H.1.8	2	97	1
AFDZ01000021_*Escherichia coli* STEC_MHI813	2	98	1
AFQH01000043_*Escherichia coli* H494	2	99	1
AFQI01000003_*Escherichia coli* TA124	2	100	1
AGSG01000200_*Escherichia coli* O103H25 str. NIPH-11060424	2	101	1
AIFQ01000025_*Escherichia coli* DEC5A	2	102	1
AIFU01000020_*Escherichia coli* DEC5E	2	103	1
AIGO01000051_*Escherichia coli* DEC9E	2	104	1
AIHK01000025_*Escherichia coli* DEC14A	2	105	1
AIHM01000060_*Escherichia coli* DEC14C	2	106	1
AJFG01000026_*Escherichia coli* SCI-07	1	107	1
AJLU01000015_*Escherichia coli* NCCP15657	2	108	1
AJMB01000011_Escherichia coli NCCP15647	2	109	1
AJPQ01000002_*Escherichia coli* AI27 1.ECAI27.1_2	2	110	1

AJWO01000049_*Escherichia coli* KD1	1	111	1
AJWP01000185_*Escherichia coli* KD2	2	112	1
AJWR01000021_*Escherichia coli* 541-1	2	113	1
AJWV01000019_*Escherichia coli* CUMT8	2	114	1
AMSK01000003_*Escherichia coli* AD30 1.ECAD30.1_3	2	115	1
AMUP01000193_*Escherichia coli* 07798 E07798.contig.187_2	2	116	1
AMVJ01000109_*Escherichia coli* 0.1288 E01288	2	117	1
CAFL01000093_*Escherichia coli* O25bH4-ST131 str. EC958	2	118	1
E1593	2	119	1
E24377A	2	120	1
E351	2	121	1
ED1a	1	122	1
EDL1943	2	123	1
F645	2	124	1
H10407	2	125	1
IAI1	2	126	1
IC18	2	127	1
IC19	2	128	1
IC21	2	129	1
IC28	2	130	1
IC33	2	131	1
IC4	2	132	1
IC47	2	133	1
IMT14973	1	134	1
IMT15009	1	135	1
IMT15010	2	136	1
IMT15150	1	137	1
IMT2111	2	138	1
IMT2121	1	139	1
IMT8103	2	140	1
IMT9087	2	141	1
MGS_124	2	142	1
MGS_73	2	143	1
MGS_89	2	144	1
NZ_ABKX01000001_*Escherichia albertii* TW07627	none	145	1
NZ_ACDM02000041_*Escherichia s* . 4_1_40B	1	146	1
NZ_ACID02000116_*Escherichia sp.* 1_1_43	2	147	1
NZ_ADTQ01000274_*Escherichia coli* MS 187-1	2	148	1
NZ_ADTY01000057_*Escherichia coli* MS 78-1	2	149	1
NZ_ADTZ01000399_*Escherichia coli* MS 116-1	2	150	1
NZ_ADUC01000346_*Escherichia coli* MS 200-1	1	151	1
NZ_ADUD01000094_*Escherichia coli* MS 196-1	2	152	1
NZ_AEME01000001_*Escherichia sp.* TW09308	1	153	1
O2	2	154	1
O42	2	155	1
O78	2	156	1
SE15	2	157	1
SMS3_5	2	158	1
TW10828	2	159	1
TW14425	2	160	1
U5070	2	161	1
W	2	162	1
