# Supplementary material for: SslE Elicits Functional Antibodies That Impair In Vitro Mucinase Activity and In Vivo Colonization by Both Intestinal and Extraintestinal Escherichia coli Strains
Source: PLoS Pathog. 2014 May 8;10(5):e1004124. doi: 10.1371/journal.ppat.1004124 (PMC4014459; doi:10.1371/journal.ppat.1004124)
Supplement: Table S3 — List of primers used in the study. (PDF) [file ppat.1004124.s007.pdf]

## Table S3. Primers used in this study

| Primer | Sequence |
|---|---|
| 1 | GGCTCTAGATTCGTAAGTCAATAATGCC |
| 2 | TTACCCGGGGCTTAAAATAGCCGCTAAA |
| 3 | TTACCCGGGTTTAAGAAATGGAATCCGG |
| 4 | TGACTCGAGGCTTATTTTTGACTGCGTA |
| 5 | GAGATATACATATGGCTAGCGATACGCCGTCTGTAGATTCTGG |
| 6 | TTTTCCTTTTGCGGCCGCTTACTCGGCAGACATCTTATGCTC |
| 7 | CTGATTTGGTATGTTGTCGGTTATAACGCTG |
| 8 | CAGCGTTATAACCGACAACATACCAAATCAG |
| 9 | GTGCGTCTCGAGCTCGGCAGACATCTTATGCTC |
| 10 | CGATCATCCGGCATTATTGACGCTAGCCATATGTATATCTCCTTCTTAAAG |
| 11 | GTGGTTCAGGATCGTCCTCCGATACGCCGTCTGTAGATTCTGG |
| 12 | GATATACATATGGCTAGCGTCAATAATGCCGGATGATCGTTAATATC |
| 13 | CTACAGACGGCGTATCGGAGGACGATCCTGAACCAC |
| 14 | AGTCAATAATGCCGGATG |
| 15 | TAATAACGCAAGTGACAAAA |
| 16 | CCACCTCTTCATTGACCAGC |
| 17 | GAGCCAGAACCTGTTCCTA |
