# Supplementary material for: SslE Elicits Functional Antibodies That Impair In Vitro Mucinase Activity and In Vivo Colonization by Both Intestinal and Extraintestinal Escherichia coli Strains
Source: PLoS Pathog. 2014 May 8;10(5):e1004124. doi: 10.1371/journal.ppat.1004124 (PMC4014459; doi:10.1371/journal.ppat.1004124)
Supplement: Methods S1 — Detailed description of the experimental procedures relative to the data reported in Fig. 6, Fig. S1 and Fig. S2. (DOCX) [file ppat.1004124.s008.docx]

**Supporting methods used in Fig. S1, Fig. S2 and Fig.6**

**Generation of IHE3034Δ*kps* strains** The IHE3034Δ*kps* was constructed by replacement of the entire the K1 *kps* gene cluster by an antibiotic resistance cassette. The upstream and the downstream regions of the *kps* gene cluster were amplified using IHE3034 chromosomal as template and cloned into the pBluescriptKS (Stratagene). The erythromycin resistance cassette was inserted between the two flanking regions in the plasmid. The resulting plasmid was used to electroporate the target strain. Lack of k1 capsule was confirmed by dot blot and cytofluorimetric analysis using the SEAM12 monoclonal antibody. The IHE3034Δ*kps*Δ*sslE* double knockout mutant was constructed by replacement of the *sslE* gene by kanamycin antibiotic resistance cassette in the IHE3034Δ*kps* strain.

**Cytofluorimetric analysis** Bacteria were grown in liquid broth and collected at the desired time points. 2,5 x 10^6^ CFU were incubated for 1h at 37°C with sera dilutions in PBS/BSA 1% in a 96-well round bottom plate. After two washes, bacteria were incubated for 45’ with the FITC-conjugated secondary antibody (Jackson ImmunoResearch Laboratories). Bacteria were fixed for 20’ in 1% paraformaldehyde, washed with PBS and acquired using the FACS Canto II. Data were analyzed by the Flow Jo software.

**ELISA assay** Flat bottom 96-well microtitre plates (MaxiSorp Nunc) were coated with 100 ng/well of purified SslE and incubated overnight at 4°C. Wells were washed with TPBS buffer (PBS/Tween 0,05%) and blocked for 1 h at 37°C with PBS/BSA 2%/Tween 0,05%. Plates were washed and incubated for 2 h at 37°C with sera serially diluted in PBS/Tween 0,05%/BSA 2%. Plates were washed and incubated with alkaline phosphatase-conjugated secondary antibody in PBS/Tween 0,05%/BSA 2% for 2h at 37°C. The development was done with 1 mg/ml of p-nitrophenyl phosphate (Sigma) in diethanolamine buffer 1M for 30’ and the reaction was stopped with NaOH 4M. Samples were carried out in duplicate and OD_405_ measurements were plotted as mean in the titration curves.

**Tissue staining** Confocal staining on tissues was performed on ileum and caecum tracts extracted from uninfected and GL53 infected mice. Tissues were fixed in 10% formalin (pH 7.2) and embedded in paraffin. Sections were cut from tissues and mounted onto slides. Samples were dewaxed and subjected to antigen retrieval before the immunofluorescence labeling. Membranes were localized with the red fluorescent Wheat Germ Agglutinin (Alexa Fluor 568-WGA, Life Technologies) and nuclei with the blue fluorescent DAPI (Life Technologies). GL53 bacteria were localized using the mouse polyclonal antibodies raised against whole cell GL53 and the green fluorescent Alexa Fluor 488-conjugated goat anti-mouse IgG (Life Technologies). Images were acquired using a 100x oil objective mounted on a Zeiss LSM710 confocal microscope.
