# Supplementary material for: SslE Elicits Functional Antibodies That Impair In Vitro Mucinase Activity and In Vivo Colonization by Both Intestinal and Extraintestinal Escherichia coli Strains
Source: PLoS Pathog. 2014 May 8;10(5):e1004124. doi: 10.1371/journal.ppat.1004124 (PMC4014459; doi:10.1371/journal.ppat.1004124)
Supplement: Text S1 — Polysialic acid capsule interferes with SslE detection on E.coli K1 IHE3034. By comparing the SslE-specific signal between IHE3034 WT and the IHE3034Δkps deletion mutant by FACS and confocal imaging analysis, we demonstrated that the K1 capsule clearly interferes with the anti-SslE antibody accessibility and recognition of the protein on the bacterial surface. (DOCX) [file ppat.1004124.s009.docx]

**Text S1.** Polysialic acid capsule interferes with SslE detection on *E.coli* K1 IHE3034

We evaluated whether the polysialic acid capsule (K1 antigen) may be responsible for masking the antigen on the bacterial surface. To test this hypothesis, we deleted the K1 *kps* gene cluster from strain IHE3034, confirming the lack of the capsule on the IHE3034Δ*kps* by FACS analysis using the SEAM12 monoclonal antibody (Fig. S1A). IHE3034 and the IHE3034Δ*kps* deletion mutant resulted in comparable growth rates in liquid media (data not shown). By comparing the SslE-specific fluorescent signal between these two strains, we demonstrated that the K1 capsule clearly interferes with the anti-SslE antibody accessibility and recognition of the protein on the bacterial surface. Indeed, anti-SslE antibody binding on the IHE3034Δ*kps* strain resulted in a stronger shift of the fluorescent signal compared to the capsulated wild-type IHE3034 strain (Fig. S1B). In order to confirm the specificity of the SslE signal in the acapsulated strain, we performed an anti-SslE antibody titration using the IHE3034Δ*kps* deleted for the *sslE* gene as a negative control (Fig. S1C). The evidence that the capsule interferes with accessibility of SslE by antibodies was further demonstrated by confocal analysis. As shown in Fig. S1D, the number of SslE positive bacteria increased from 3% on wild-type IHE3034 (Fig. 1) to roughly 100% on the IHE3034Δ*kps* (Fig. S1D). As expected, the IHE3034Δ*kps*Δ*sslE* strain was negative for SslE expression (Fig. S1E). We then conclude that SslE is efficiently exported to the surface of *E. coli* IHE3034, but that the capsule may mask it in the *in vitro* conditions used. However, we assume that SslE accessibility on K1 IHE3034 during infection may be modulated by several factors including capsule expression. Indeed, the K1 capsule plays a role during host-bacteria interactions by regulating the expression from extensive levels to complete shutdown in sites that may afford protection from immune attack. It is likely that SslE exposure and accessibility *in vivo* may be dependent on the dynamic masking role due to the capsule in K1 *E. coli*.
