# Supplementary material for: SslE Elicits Functional Antibodies That Impair In Vitro Mucinase Activity and In Vivo Colonization by Both Intestinal and Extraintestinal Escherichia coli Strains
Source: PLoS Pathog. 2014 May 8;10(5):e1004124. doi: 10.1371/journal.ppat.1004124 (PMC4014459; doi:10.1371/journal.ppat.1004124)
Supplement: Text S2 — IgG and IgA antibody response following SslE immunization. We observed that subcutaneous immunization of rabbit with recombinant SslE generated a high response in terms of IgG, while IgA values were low. (DOCX) [file ppat.1004124.s010.docx]

**Text S2.** IgG and IgA antibody response following SslE immunization

We performed enzyme-linked immunosorbent assays (ELISAs) on serum derived from rabbit immunized with recombinant SslE (used in the in vitro inhibition of bacterial crossing through a mucin-gel matrix) and on sera from mice tested in protection experiments (*i.e.*, in the mouse model of intestinal colonization). As showed in Fig. S2A and B, subcutaneous immunization of rabbit with SslE generated a high response in terms of IgG, while IgA values were low. This is not surprising, given that IgA responses to subcutaneous immunization are generally very low. Serum derived from rabbit to which Freund’s adjuvant alone was administered was used as negative control (Fig. S2A-B). On the other hand, we analyzed a pool of sera derived from 16 mice intranasally immunized with recombinant SslE. Titration curves of pooled sera demonstrated the induction of good antibody response in terms of both IgG and IgA in mice (Fig. S2C and D). In this case, control sera were relative to mice immunized with saline alone.
