# Supplementary material for: SslE Elicits Functional Antibodies That Impair In Vitro Mucinase Activity and In Vivo Colonization by Both Intestinal and Extraintestinal Escherichia coli Strains
Source: PLoS Pathog. 2014 May 8;10(5):e1004124. doi: 10.1371/journal.ppat.1004124 (PMC4014459; doi:10.1371/journal.ppat.1004124)
Supplement: Text S3 — Polyclonal antibodies against the truncated C-SslE impair E. coli translocation through a mucin matrix. We postulated that antibodies against the N-terminal portion of the protein lacking the M60-like zinc-metalloprotease motif are still able to impair E. coli translocation through the mucin matrix, suggesting an indirect inhibitory activity. (DOCX) [file ppat.1004124.s011.docx]

**Text S3.** Polyclonal antibodies against the truncated C-SslE impair *E. coli* translocation through a mucin matrix

We have cloned, expressed and purified a truncated SslE fragment lacking the M60-like zinc-metalloprotease HEXXHX(8,24)E motif (Fig. S3A). Antisera derived from mice immunized with the truncated form of SslE (named C-SslE) were tested for their ability to interfere with the bacterial crossing in the quantitative mucin-gel matrix degradation assay. As shown in Fig. S3B, antibodies against the N-terminal portion of the protein lacking the M60-like zinc-metalloprotease motif are still able to impair *E. coli* translocation through the mucin matrix. These data support the hypothesis that the effect of anti-SslE polyclonal antibodies reported in Fig. 3 may be due to an indirect inhibitory activity.
